# LETM-domain containing 1 (LETMD1) protects against obesity via enhancing UCP1-independent energy expenditure in human beige adipocytes

**DOI:** 10.7150/thno.104568

**Published:** 2025-01-02

**Authors:** Jiaxing Liu, Ying Cheng, Qing Liu, Qiaoyun Long, Shiqing Liang, Wei Sun, Kerry M. Loomes, Xuefei Gao, Bin Lin, Xingguo Liu, Donghai Wu, Hannah Xiaoyan Hui

**Affiliations:** 1 School of Biomedical Sciences, The Chinese University of Hong Kong, Hong Kong, China.; 2 Guangdong Provincial Key Laboratory of Stem Cell and Regenerative Medicine, Guangzhou Institutes of Biomedicine and Health, Chinese Academy of Sciences, Guangzhou, China.; 3 China-New Zealand Joint Laboratory on Biomedicine and Health, Guangzhou, China.; 4 School of Biological Sciences & Maurice Wilkins Centre, University of Auckland, Auckland, New Zealand.; 5 School of Biomedical Sciences, Southern Medical University, Guangzhou, China.; 6 Key Laboratory of Structure-Based Drug Design and Discovery of Ministry of Education, Wuya College of Innovation, Shenyang Pharmaceutical University, Shenyang 110016, China.; 7 GIBH-CUHK Joint Research Laboratory on Stem Cell and Regenerative Medicine, Guangzhou Institutes of Biomedicine and Health, Chinese Academy of Sciences, Guangzhou, China.; 8 CUHK-GIBH Joint Research Laboratory on Stem Cell and Regenerative Medicine, The Chinese University of Hong Kong, Hong Kong, China.

**Keywords:** LETMD1, energy expenditure, UCP1, beige adipocyte, SP-8356

## Abstract

**Rationale:** Brown and beige adipocytes are specialized fat cells that dissipate energy in the form of heat, and hold therapeutic potential for obesity and metabolic diseases. Although in the classical viewpoint brown and beige adipocytes dissipate energy solely via uncoupling protein 1 (UCP1), emerging evidence suggests the importance of non-canonical UCP1-independent energy expenditure in regulating energy expenditure, especially in human beige adipocytes. Leucine zipper-, EF-hand-containing transmembrane protein 1 domain containing 1 (LETMD1) was recently identified as a key protein in maintaining UCP1 expression and the thermogenic activity of brown adipocytes in animal models. But the exact function of LETMD1 and its mechanism of action in human beige adipocytes are unclear.

**Methods:** We tested the function of LETMD1 in human induced pluripotent stem cell (hiPSC)-derived beige adipocytes *in vitro* in both wildtype (WT) and *UCP1* knockout (KO) background. Furthermore, human beige adipocytes harboring a doxycycline-inducible *LETMD1* expression cassette were transplanted to NOD/SCID mice and the function of LETMD1 in human beige adipocytes was evaluated in the *in vivo* setting. RNA-Seq was conducted in normal and *LETMD1*-overexpressing human beige adipocytes to examine the genes and pathways regulated by LETMD1. Using a knock-in human iPSC line, a preclinical small molecule compound library was screened for compounds increasing *LETMD1* expression in human beige adipocytes. The effects of the compound in inducing *LETMD1* and UCP1-independent energy expenditure in beige adipocytes were examined *in vitro* and in animal models.

**Results:** LETMD1 plays an essential role in engaging energy dissipation, in a manner independent of UCP1, in human beige adipocytes. Transplantation of *LETMD1*-overexpressing human beige adipocytes improved whole-body metabolism of the recipient mice independent of UCP1. Mechanistically LETMD1 enhances the transcription of *PPARGC1A*, a key regulator of mitochondrial biogenesis. The expression of genes related to UCP1-independent energy expenditure, including creatine futile cycle, was also stimulated upon *LETMD1* overexpression. Using *LETMD1* reporter human beige adipocytes, SP-8356 was identified as a compound significantly increasing *LETMD1* expression. Oral administration of SP-8356 induced genes related to UCP1-independent energy expenditure in beige adipocytes, and counteracted body weight gain and metabolic disorders in mice.

**Conclusion:** Increased LETMD1 action, either genetically or pharmacologically, enhances the non-canonical UCP1-independent energy expenditure in beige adipocytes.

## Introduction

Adipose tissue can be classified into two functionally distinct tissues: white adipose tissue (WAT), which is responsible for storing energy 1, and brown adipose tissue (BAT), which consumes energy 2. A third type of adipose tissue, the beige fat, also called the “brite” (i.e. “brown in white”) adipocytes, is found in WAT and mobilized in response to appropriate stimuli 3. Multiple proof-of-concept studies have shown that activation of beige adipocytes confers protection against obesity, metabolic and cardiovascular diseases.

Upon cold exposure, the quiescent beige adipocytes which are scattered within the WAT are reprogrammed extensively, so as to adopt a brown-like, metabolically active phenotype 4. In particular, the activated beige adipocytes are rich in smaller lipid droplets (multilocular) and functional mitochondria, and abundantly express UCP1, which is a key effector of energy dissipation by uncoupling the electron transport chain to ATP production 1. Importantly, emerging evidence unravels that beige adipocytes also exert their metabolic protective actions in alternative mechanisms independent of UCP1, including enhancing futile calcium cycling and creatine cycling 5, 6. Most recently, ATP5K+ beige adipocytes which are devoid of UCP1 expression were detected in human and mice adipose tissue, and they engage in UCP1-independent energy expenditure via creatine futile cycling 7, 8. Although the importance of UCP1 in maintaining adipose energy expenditure in mammals is well established, and in adult human the metabolically active brown-like adipose tissue was detected 9-11, recent single nuclei sequencing (sn-Seq)-based analysis in human WAT barely detected UCP1+ adipocytes 12, suggesting that UCP1-independent energy dissipation might play an even more dominant role compared to the classical UCP1-dependent mechanism in human beige adipocytes. However, the efficient strategies to stimulate UCP1-independent energy dissipation in human beige adipocytes are still lacking.

LETMD1 was initially identified as a mitochondrial outer membrane protein and is believed to play an essential role in BAT thermogenesis 13. The expression of *LETMD1* is highest in the BAT in mice and is significantly elevated after cold stimulation 14. *Letmd1* gene knockout (KO) mice are unable to adapt to acute cold stimulation, and exhibit impaired mitochondrial function, whitening of BAT and a significant decrease in metabolism 14-18. This is achieved via modulating various cellular mechanisms within the brown adipocyte, such as regulating mitochondrial protein import and synthesis 16, 17, or directly acting as the co-regulator of other transcription factor 18.

The functional role of LETMD1 in beige adipocytes, especially in human beige adipocytes as well as the potential clinical applications of LETMD1 in human beings, however, is less well understood. In this study, we demonstrated that LETMD1 increases the energy expenditure of human beige adipocytes in a UCP1 independent fashion. In addition, we found that transplanted human beige adipocytes overexpressing *LETMD1* ameliorate obesity and decrease symptoms of metabolic diseases in recipient animals, suggesting a potentially effective cellular therapy. Furthermore we identified a small molecule compound, SP-8356, that potently increases the expression of *LETMD1* in both human and mouse beige adipocytes. Oral administration of SP-8356 protects against high fat diet (HFD) induced-obesity and metabolic diseases in mice accompanied with an increase of UCP1-independent thermogenic gene expression in beige adipocytes.

## Methods

### Culture of hiPSC

HiPSCs were cultured on SNL feeder layers and maintained in EPSCM: DMEM/F12 (Gibco, U.S.), 1X NEAA (Thermo Fisher Scientific, U.S.), 1X Normocin (InvivoGen, California), 1X Pen-strep-glutamine (Thermo Fisher Scientific, U.S.), 10 ng/ml of recombinant human LIF protein (Sino Biological, China), B27 supplement (Thermo Fisher Scientific, U.S.), N2 supplement (Thermo Fisher Scientific, U.S.), 50 µg/ml of ascorbic acid (Sigma, U.S.), 100 µM of 2-mercaptoethanol (Sigma, U.S.), 1 µM of CHIR99021(TARGETMOL, Boston), 2.5 µM of XAV939 (TARGETMOL, Boston), 0.1 µM of A-419259 (TOCRIS, UK), 2% KnockOut Serum Replacement (KSR) (Gibco, U.S.). SNL feeder cells were grown to confluency and inactivated by mitomycin C (Sigma-Aldrich, U.S.) and were seeded on 0.1% gelatin-coated plates cultured in Dulbecco's Modified Eagle Medium (DMEM) (Gibco, U.S.) with 10% fetal bovine serum (FBS) (Gibco, U.S.). HiPSCs were passaged every 3-4 days at a ratio of 1:8 by dissociating into single cells using StemPro Accutase (Thermo Fisher Scientific, U.S.) for 3-5 min. 5 μM of Y27632 (STEMCELL Technologies, Canada) was added into the culture medium for each passage.

### Differentiation of hiPSCs to beige adipocytes

The method for the differentiation of human beige adipocytes from hiPSCs was conducted based on a previous publication 19. In the initial stage, 10 ng/ml of bone morphogenic protein-4 (STEMCELL Technologies, Canada) and 25 ng/ml of activin A (STEMCELL Technologies, Canada) were used from day 0 to day 4 to induce mesodermal differentiation. From day 4 to day 10, 10 μg/ml of insulin (Thermo Fisher Scientific, U.S.), 500 μmol/L of isobutylmethylxanthine (IBMX) (Sigma, U.S.), 1 μM of dexamethasone (Sigma, U.S.), and 50 μM of indomethacin (Sigma, U.S.) were used to induce differentiation of the mesodermal progenitors into adipocyte progenitors. From day 10 to 20, 1 μg/ml of insulin was used to culture the differentiating human beige adipocytes.

### Animal studies

10-12 week old female NOD/SCID mice were purchased from the Laboratory Animal Services Centre of the Chinese University of Hong Kong and were fed with either standard chow diet (STC; D12450B, Research Diets Inc.) or HFD (D12451, Research Diets Inc.). *Ucp1* KO mice were purchased from Shanghai Model Organisms Center, Inc. All animal procedures were approved by the University's Animal Experimentation Ethics Committee (AEEC) and followed the principles and guidelines set by the Animal Holding Core, School of Biomedical Sciences, The Chinese University of Hong Kong. All animals were maintained on a 12 h light-dark cycle under the controlled environmental settings (22 °C to 24 °C), with *ad libitum* access to sterilized water and food. The male C57BL/6J mice were fed with SP-8356 (50 mg/kg/day) in drinking water as described previously 20. Dosage was determined based on average daily water consumption (6 mL/day for each mouse).

### Transplantation of human beige adipocytes

Inducible* LETMD1* expressing hiPSCs were differentiated into beige adipocytes respectively, followed by transplantation into the hindlimb muscles of NOD/SCID mice. A total of 2.4 x 10^6^ beige adipocytes were transplanted into each recipient. After recovery for 7 days, the mice were gavaged with 200 µl of doxycycline solution (10 mg/ml) daily to activate *LETMD1* expression in the transplanted cells. PBS was used as the vehicle control.

### Indirect calorimetry

Promethion Metabolic System from Sable (Nevada, USA) was used to measure the metabolic rate of the mice. After transplantation of the beige adipocytes and treatment with doxycycline solution daily for five days, mice were singly housed in the metabolic cages for three days with free access to sterilized water and food, with a 12 h light-dark cycle under controlled environmental settings (22 °C to 24 °C). The metabolic variables were recorded by the Promethion software, MetaScreen, and ExpeData.

### Glucose tolerance test (GTT)

Mice were switched to clean cages and fasted for 16 h. The next day, 20% D-glucose (w/v) at a dose of 2 g/kg was intraperitoneally injected, and blood glucose levels were measured at 0, 15, 30, 45, 60, and 90 min with the One-touch Glucose Meter (LifeScan, California).

### Hematoxylin and Eosin (H&E) staining

Mice were sacrificed, and then the muscle samples were isolated and fixed in 4 % formaldehyde solution (Sigma, U.S.) for 24 h. Tissues were then dehydrated using a Thermo Excelsior ES tissue processor, followed by paraffin embedding using Epredia HistoStar Embedding center, and sectioned at 5-μm-thick using Leica RM2235 Rotary Microtome. The sections were then de-paraffinized and rehydrated before H&E staining, and images were taken with the Nikon Ti2-E Inverted Fluorescence Microscope.

### Immunofluorescence staining

Immunofluorescence analysis was performed using the paraffin sections. Pretreatment of the sections, including de-paraffinization and rehydration, was carried out using the same procedures as for H&E staining (first eight steps). Antigen retrieval was then performed on the sections using the Thermo PT Module Antigen Retriever by boiling in sodium citrate buffer at 98 °C for 20 min. After blocking the sections with 3% BSA (in PBS) for 1 h at room temperature in a humidity chamber, the sections were washed with TBST for 2 min and incubated with primary antibodies against LETMD1 (Invitrogen, U.S.) and Perilipin 1 (Novus Biologicals, LLC, U.S.) at 4 ℃ overnight. The next day, the sections were washed three times with TBST (10 min for each wash) and incubated with anti-rabbit IgG (H+L) cross-adsorbed secondary antibody (Invitrogen, U.S.) for 1 h in a humidity chamber at room temperature with protection from light. After washing three times with TBST, the sections were mounted on cover slides with antifade mounting medium with DAPI. Images were taken using the Nikon Ti2-E Inverted Fluorescence Microscope.

### Construction of UCP1 KO and LETMD1 KO hiPS single cell lines using the CRISPR-Cas9 genome editing system

Two single guide RNAs (sgRNA) that we designed to target exon 2 of the *UCP1* gene were cloned into the pSpCas9 (BB)-2A-GFP (PX458, www.addgene.org) vector. Similarly, two sgRNAs targeting exon 3 of the *LETMD1* gene were designed and integrated into the same plasmid vector. HiPSCs were cultured in one well of a 6-well plate and transfected with 4 μg of the indicated plasmids using LipofectamineTM 3000 reagent (InvitrogenTM, cat. no. L3000001) following the manufacturer's instructions. 48 h after transfection, the GFP-positive cells were sorted by the BD FACSAria Fusion Cell sorter. The single clones were picked 4-5 days later. After each cell line was harvested, cell aliquots were either stored or used for genomic DNA extraction.

### Genomic DNA extraction and PCR

The genomic DNA was extracted using RELIAPREP(TM) BLOOD GDNA MINIPREP SYSTEM (Bio-Gene Technology, Australia) according to the instructions. The DNA samples were mixed with the given primers and 2xEs Taq MasterMix (Dye) (Cwbio, China), and then subjected to PCR amplification according to the specific procedure. Subsequently, the PCR products were electrophoresed using a 2% agarose gel. The gel was observed by an Ultraviolet transilluminator (Bio-Rad ChemiDoc Imaging System), and the PCR products were sent to Sanger sequencing.

### Seahorse metabolic assays

HiPSCs were dissociated with accutase and then counted. Cells were then grown at 5.9 × 10^6^ cells per cm^2^ in Agilent SeaHorse XFe96 Cell Analyzer-specific microtiter plates. After hiPSCs were differentiated into beige adipocytes as previously described, the medium was replaced with a sodium bicarbonate free medium and incubated for 1 h in a CO_2_-free incubator. Oligomycin (15 μM), FCCP (10 μM), and rotenone/antimycin A (2 μM each) were sequentially added to the cells at the indicated time points. For evaluating the contribution of creatine cycle in human beige adipocytes, the cells were pretreated with 2mM of β-GPA for 24 h before Seahorse analysis.

### RNA extraction and real-time PCR

Total RNAs from cells were extracted with RNAiso Plus reagent (TAKARA BIO INC., Japan) following the manufacturer's instructions. The RNA concentration and purity were measured by the Nano-drop microspectrophotometer. 1μg of total RNA for each sample was reverse-transcribed into complementary DNA (cDNA) following the instruction manual of the PrimeScript RT reagent Kit. Real-time PCR was performed using the TB Green Premix Ex Taq (TAKARA BIO INC., Japan) and ROX Reference Dye (TAKARA BIO INC., Japan). Primer sequences for real-time PCR are listed in the Supplementary Table 1. Real-time PCR was conducted in triplicate using the ABI ViiA7 Real-Time PCR System (Applied Biosystems, Foster City, CA, U.S.). The expression level of housekeeping gene β-actin was used to normalize the relative expressions of the target genes. The relative expression of all genes was analyzed using the 2^-ΔCT^ method.

### Western blot analysis

Cells were washed twice with PBS and lysed by RIPA buffer. Protein concentrations were measured by the BCA protein assay (FD2001, HANGZHOU FUDE BIOLOGICAL TECHNOLOGY CO., LTD, China). The lysates were resolved by SDS-PAGE gel electrophoresis (HANGZHOU FUDE BIOLOGICAL TECHNOLOGY CO., LTD, China) and then transferred to the PVDF membrane (Bio-Rad Laboratories Inc., U.S.) using the electrophoretic transfer systems for wet blotting methods. Each membrane was blocked for 1 h with 5% non-fat milk and incubated with a specific primary antibody overnight at 4 ℃, with anti-LETMD1 (1:1000) (Invitrogen), anti-UCP1 (1:1000) (Abcam), anti-HSP90 (1:1000) (Cell signalling technology), followed by washing 3 times for 15 min each with 0.1% PBS-Tween buffer. The membrane was then incubated with a secondary antibody for 1 h and was detected by ECL reagents (Bio-Rad Laboratories Inc., U.S.). Images were taken with the Bio-Rad ChemiDoc MP Imaging System, and the amounts of the target proteins were normalized to their respective internal references using Image J analysis software.

### Statistical analysis

All data were presented as mean with SD, and analysis was performed using SPSS (version 16.0, SPSS Inc., U.S.). Statistical significance was calculated by unpaired two-tailed Student's *t* test, ANOVA or analysis of covariance (ANCOVA) (*P < 0.05, **P < 0.01, ***P < 0.001, and ****P < 0.0001).

## Results

### LETMD1 plays an essential role in maintaining energy expenditure in human beige adipocytes

**Figure 1:**
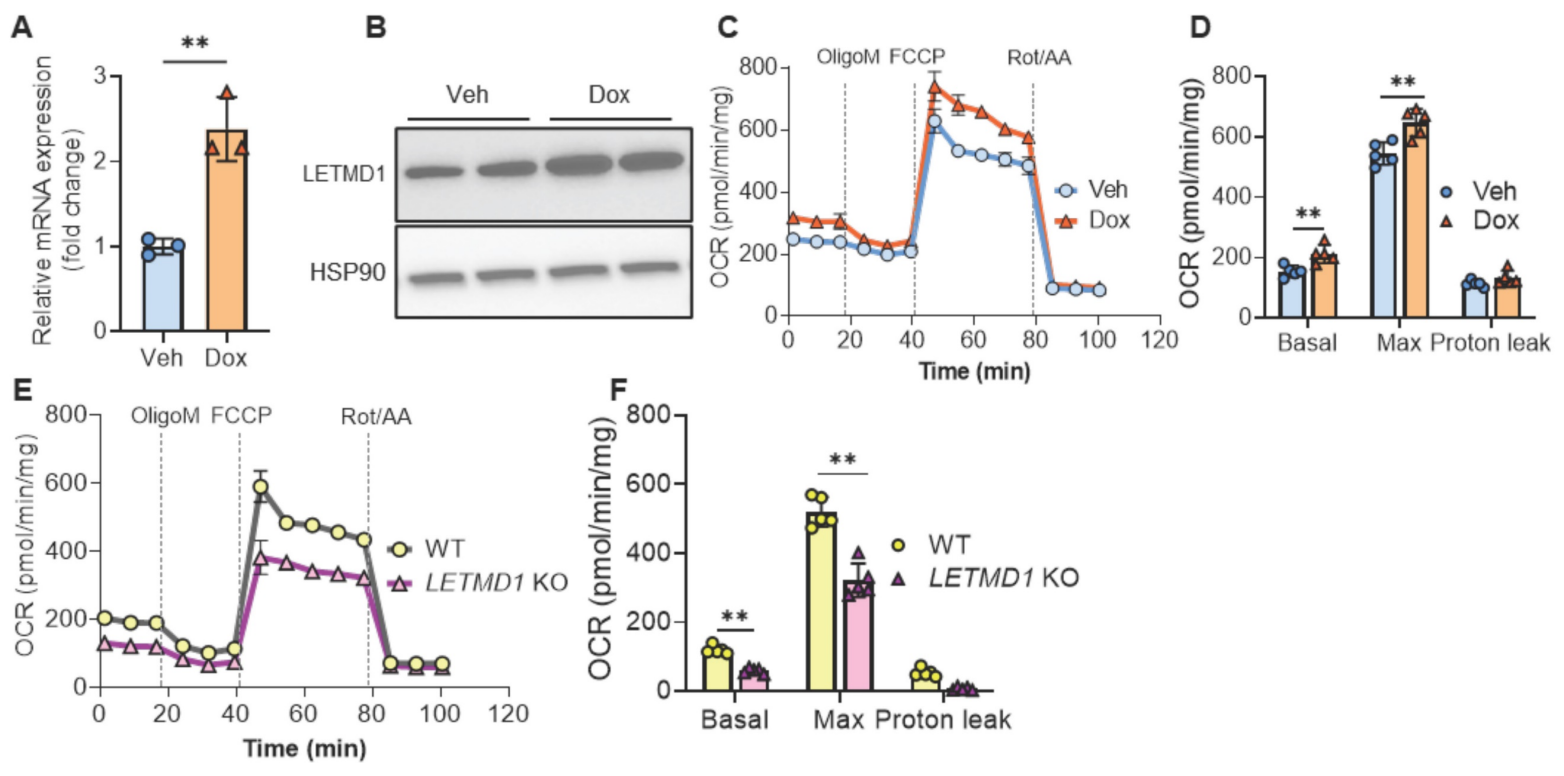
** LETMD1 enhances energy expenditure in human beige adipocytes. (A)-(D)** HiPSC line with doxycycline (Dox)-inducible *LETDM1* expression was established and differentiated into beige adipocytes and treated with doxycycline or vehicle (Veh) for 24 h before analysis. **(A)** Relative mRNA expression of *LETMD1* gene in beige adipocytes. n = 3 for each group.** (B)** Western blot analysis of LETMD1 in beige adipocytes. **(C)** Mitochondrial stress test to measure the OCR in beige adipocytes. n = 5. **(D)** Basal, maximal and proton leak respiration calculated from **(C)**. n = 5. **(E)-(F)** *LETMD1* KO hiPSC line was generated and differentiated to beige adipocytes for analysis. **(E)** OCR in beige adipocytes. n = 5. **(F)** Basal, maximal and proton leak respiration calculated from **(E)**. n = 5. All data are presented as mean with SD. Statistical significance was calculated by unpaired two-tailed Student's *t* test (*P < 0.05, **P < 0.01).

To examine the biological function of LETMD1 in human beige adipocytes, we adopted a previously published protocol to differentiate hiPSCs to functional beige adipocytes 19 (Figure S1A). Successful differentiation was validated by accumulation of lipid droplets, the induction of stage markers at different time points at both mRNA and protein levels (Figure S1B-I). An *LETMD1*-inducibly expressed hiPSC line was generated (Figure S2A), which upon treatment with doxycycline, increased expression of LETMD1 at mRNA and protein levels, respectively (Figure 1A-B). Importantly, overexpression of LETMD1 led to an increase in both basal and maximal oxygen consumption rate (OCR) in human beige adipocytes (Figure 1C-D). These increases were mainly attributed to coupled respiration whereas the uncoupled respiration was not changed by LETMD1 overexpression (Figure 1D). Conversely, CRISPR/Cas9-mediated deletion of the *LETMD1* gene mitigated basal and maximal cellular respiration (Figure 1E-F and Figure S2B-C). Therefore both gain-of-function and loss-of-function experiments demonstrated that LETMD1 plays a pivotal role in stimulating the energy expenditure in human beige adipocytes, but via a non-canonical, coupled mechanism.

### LETMD1 enhances mitochondrial biogenesis in human beige adipocytes

**Figure 2:**
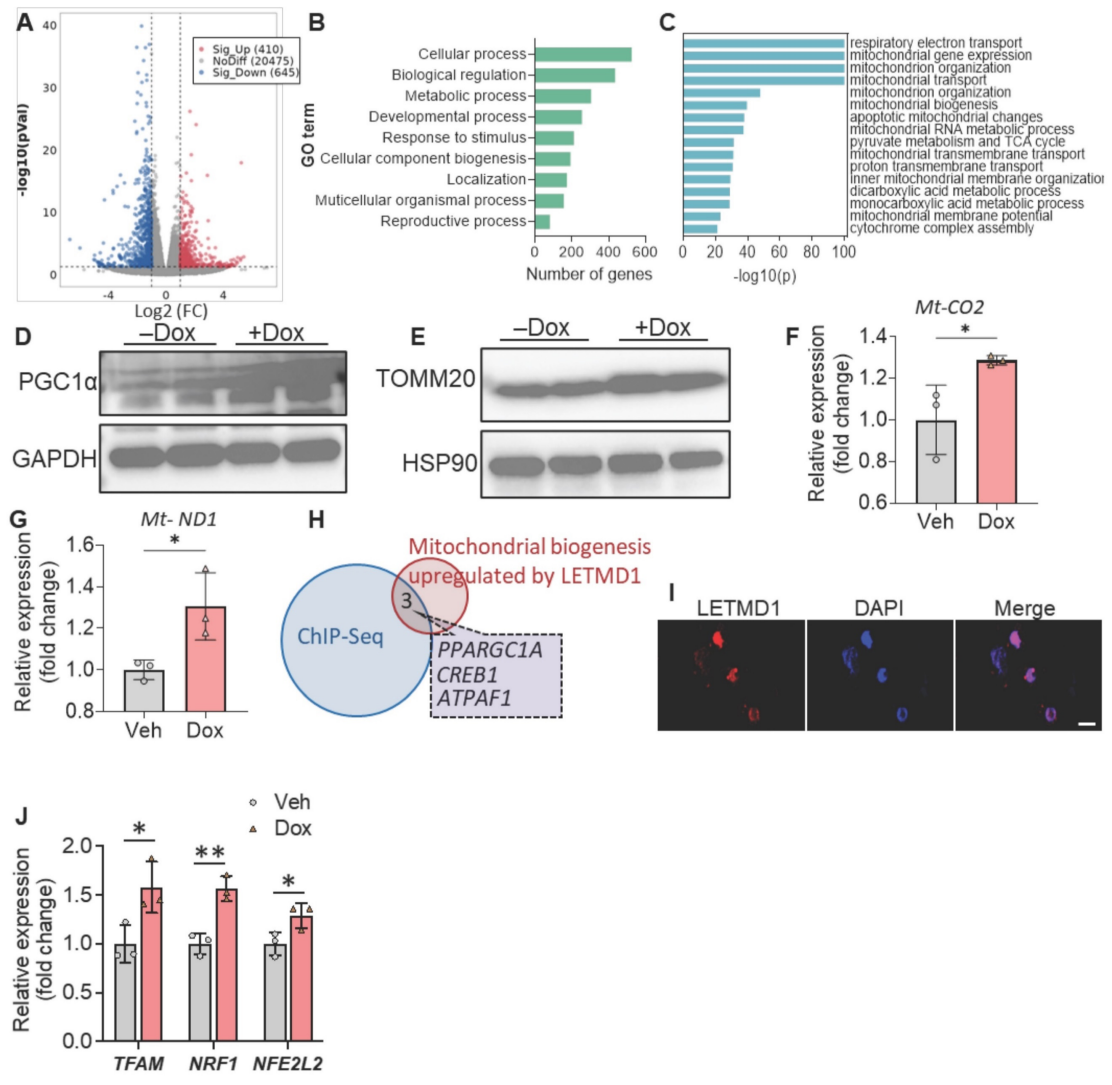
** LETMD1 enhances mitochondria biogenesis in human beige adipocytes.** Doxycycline-inducible *LETMD1* hiPSCs were differentiated into beige adipocytes and treated with doxycycline or vehicle for 24 h before analysis. **(A)-(C)** Bulk RNA-Seq of human beige adipocytes treated with doxycycline or vehicle. **(A)** Volcano plot (Dox vs. Veh). **(B)** GO annotation analysis. **(C)** Metascape analysis on the genes included in the “metabolic process” in the GO annotation in **(B)**. **(D)-(E)** Western blot of PGC1α **(D)**, and TOMM20 **(E)**. **(F)-(G)** The relative level of mitochondrial DNA, *Mt-CO2* gene** (F)** and *Mt-ND1* **(G)**.** (H)** Venn diagram of genes showing downregulated binding in ChIP-Seq data and mitochondrial biogenesis related genes upregulated by *LETMD1* overexpression in **(C)**. **(I)** Representative image of immunofluorescence staining of LETMD1. Scale bar = 10 µM. **(J)** Realtime PCR measuring the mRNA expression of key transcription factors. n = 3. All data are presented as mean with SD. Statistical significance was calculated by DEseq2 **(A)** or unpaired two-tailed Student's *t* test **(F-G and J)** (*P < 0.05, **P<0.01).

We next proceeded to investigate the mechanism by which LETMD1 stimulates energy expenditure in human beige adipocytes. RNA-Seq analysis identified 410 and 645 genes, upregulated and downregulated, respectively, upon induction of LETMD1 expression in human beige adipocytes (Figure 2A). Through gene ontology (GO) annotation analysis, it was found that LETMD1 overexpression significantly affected multiple metabolic processes (Figure 2B). Metascape analysis showed that the pathways upregulated by LETMD1 overexpression were mainly enriched for mitochondrial function, such as electron transport and the TCA cycle, and mitochondrial biogenesis as well (Figure 2C). In particular, we found that *PPARGC1A* (encoding peroxisome proliferator-activated receptor-γ coactivator 1-α, PGC1α) is among the upregulated genes (Supplementary Table 3); the increase in PGC1α was validated by Western blotting analysis (Figure 2D). In addition, expression of mitochondrial outer membrane protein, TOMM20, and the copy number of mitochondrial DNA (mtDNA) were significantly elevated upon overexpression of LETMD1 (Figure 2E-G), whereas expression of oxidative phosphorylation related proteins remained unchanged (Figure S3). These findings suggest that LETMD1 primarily enhances mitochondrial biogenesis in human beige adipocytes.

Although LETMD1 was originally described as a mitochondrial protein, LETMD1 reportedly also localizes to the nucleus of mice brown adipocytes, where it directly interacts with the transcriptional coregulator and chromatin remodeler, Brg1/Smarca4 18. Consistent with this finding, immunofluorescence staining results showed that LETMD1 protein was present in the nucleus of human beige cells (Figure 2I). To look for those genes whose transcription was directly regulated by LETMD1, genes upregulated by LETMD1 (410 genes) were overlapped with genes showing reduced binding with Brg1 in *Letmd1* KO brown adipocytes in chromatin IP sequencing (ChIP-seq) data 18. Three genes were identified, one of which was *PPARGC1A* (Figure 2H). These results indicated that the transcription of *PPARGC1A* is directly regulated by LETMD1.

PGC1α is the master regulator of mitochondrial biogenesis and function, mainly through inducing the expression of three key transcription factors, i.e., *NRF1, NFE2L2,* and* TFAM* 21, 22. Indeed we found that overexpression of LETMD1 led to significantly increased expression of all these three key genes (Figure 2J). Collectively these results demonstrated that LETMD1 enhances mitochondrial biogenesis through directly regulating the transcription of *PPARGC1A* gene in human beige adipocytes.

### LETMD1 engages UCP1-independent energy expenditure

**Figure 3:**
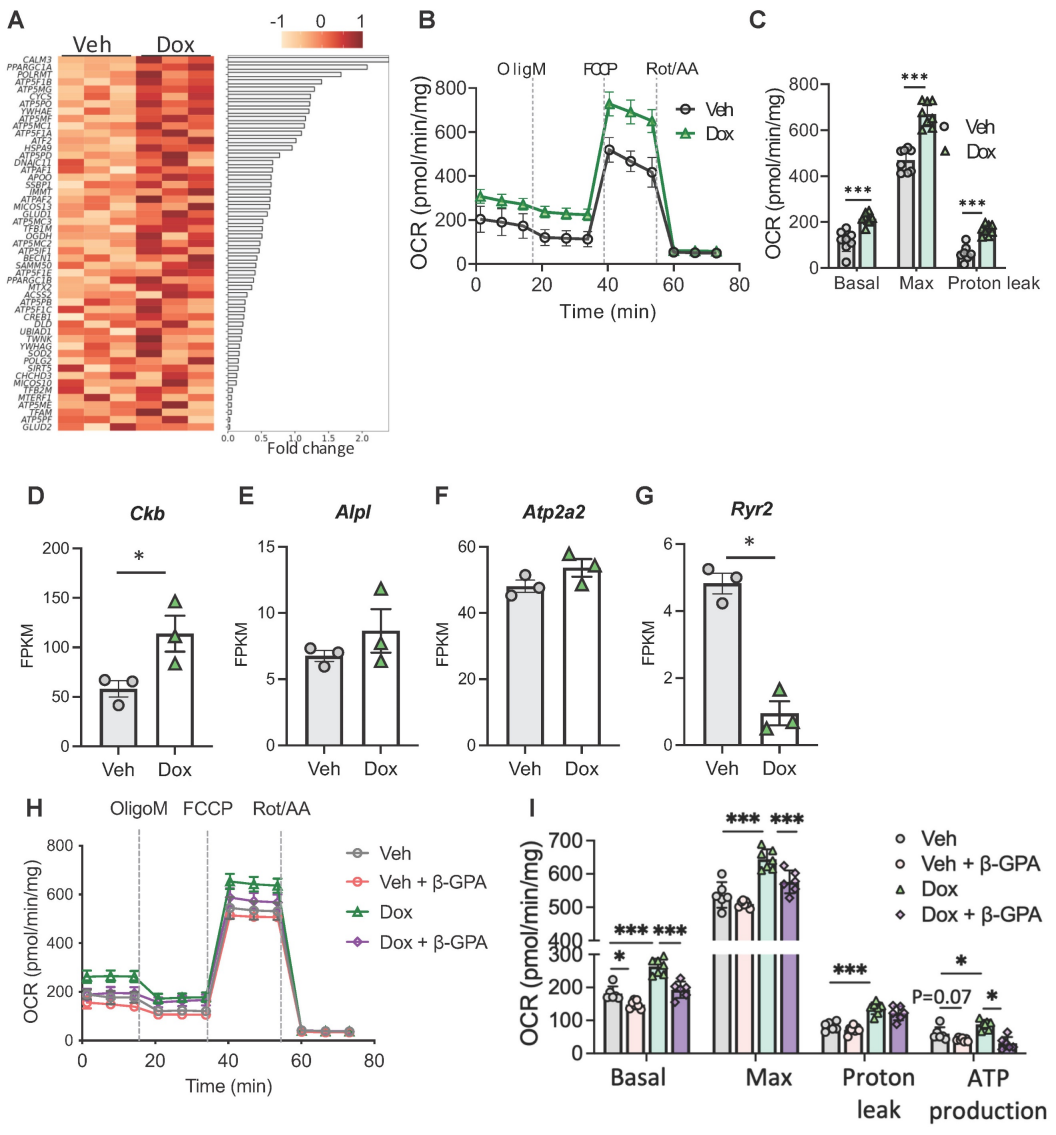
** LETMD1 induces UCP1-independent energy expenditure. (A)** Doxycycline-inducible *LETMD1* hiPSCs were differentiated into beige adipocytes and treated with doxycycline or vehicle solution for 24 h before bulk RNA-Seq. Heatmap of differentially expressed genes (DEG).** (B)-(C)** *UCP1* KO hiPSC line with doxycycline-inducible *LETDM1* expression was differentiated into beige adipocytes, followed by treatment with doxycycline or vehicle for 24 h. **(B)** OCR measured by the Seahorse Mitochondrial Stress Test. n = 8. **(C)** Basal, maximal and proton leak respiration calculated from **(B)**. n = 8. **(D)-(G)** mRNA expressions of genes in UCP1-independent energy expenditure measured by bulk RNA-Seq. n = 3. FPKM, Fragments Per Kilobase of transcript per Million mapped reads. **(H)-(I)** Doxycycline-inducible *LETMD1* hiPSCs were differentiated into beige adipocytes and treated with doxycycline or vehicle solution and β-GPA (2mM) for 24 h, before OCR was examined. n = 6-7. All data are presented as mean with SD. Statistical significance was calculated by unpaired two-tailed Student's *t* test **(C)**, DEseq2 **(D-G)**, two-way ANOVA **(I)**. (*P < 0.05, **P<0.01, ***P<0.001).

Interestingly, among the genes that were upregulated by LETMD1 several *ATP5* genes, which encode the subunits of mitochondrial ATP synthase, were upregulated in LETMD1 overexpressing beige adipocytes (Figure 3A). A recent study reported that ATP5K+ beige adipocytes are present in human WAT and are energy consuming beige adipocytes acting in a UCP1-independent manner 8. This finding suggests that LETMD1 likely also enhances energy expenditure via UCP1-independent mechanisms, which is consistent with our initial finding that LETMD1-evoked energy expenditure is achieved via a coupled manner (Figure 1C).

To support this hypothesis, we first constructed *UCP1* KO hiPS single-cell clones using CRISPR-Cas9 technology. The successful deletion of *UCP1* gene was verified by PCR, Sanger DNA sequencing and Western blotting analysis (Figure S4A-C). Next the doxycycline-inducible *LETMD1* expression cassette was introduced into the *UCP1* KO hiPSCs using the same strategy as shown in Figure S2. Although the deletion of *UCP1* led to an increased expression of *LETMD1* compared to the WT beige adipocytes (Figure S4D), doxycycline still enhanced LETMD1 expression in *UCP1* KO human beige adipocyte (Figure S4E-F), demonstrating successful construction of *UCP1* KO-*LETMD1* inducibly expressed human beige adipocytes.

Importantly, overexpression of *LETMD1* still stimulated energy expenditure in *UCP1* KO background (Figure 3B-C). Consistent with these findings, expression of Creatine kinase B (*CKB*), the gene involved in mitochondrial creatine futile cycling 6, was significantly upregulated in human beige adipocytes overexpressing *LETMD1* (Figure 3D). Alkaline phosphatase (*ALPL*), also involved in creatine futile cycling, showed a trend of increase (Figure 3E). By comparison, other reported UCP1-independent thermogenesis related genes such as *ATP2A2* and *RYR2,* which involve calcium futile cycling between cytosol and ER, were either not altered or downregulated (Figure 3F-G). To further examine the downstream mechanism mediating the effect of LETMD1, we treated *LETMD1* overexpressing human beige adipocytes with β-guanidinopropionic acid (β-GPA), a creatine analog to inhibit creatine futile cycling 23. Inhibition of creatine futile cycling significantly mitigated the OCR induced by *LETMD1* overexpression, especially the ATP production-associated OCR (Figure 3H-I), indicating that creatine futile cycling is a major thermogenic mechanism of LETMD1 action. Collectively, these results demonstrate that LETMD1 enhances energy expenditure in human beige adipocytes via creatine futile cycling in UCP1-independent manner.

### Transplantation of LETMD1-overexpressing human beige adipocytes improves metabolic disorders in mice

**Figure 4:**
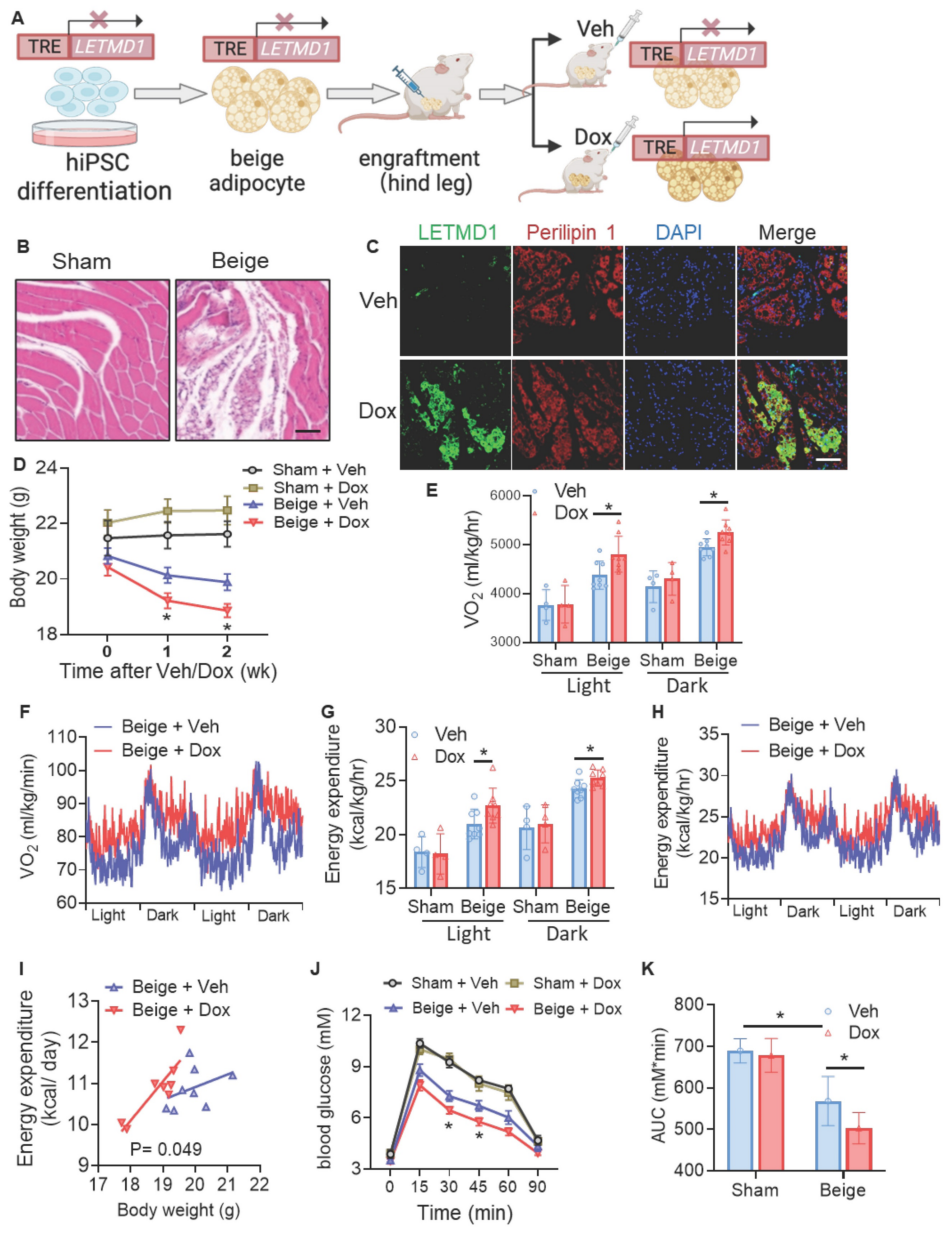
** Induction of *LETMD1* in beige adipocytes improves metabolism in mice.** HiPSC with TRE-*LETMD1* cassette was differentiated *in vitro* to beige adipocytes, followed by transplantation to NOD/SCID mice. After recovery, the mice were orally gavaged with doxycycline (Dox) or vehicle (Veh) for two weeks. **(A)** Schematic graph of the experiment. **(B)** H&E staining to visualize the transplanted beige adipocytes in the hind leg of the recipient mice. Scale bar = 100 μM. **(C)** Immunofluorescence staining of LETMD1 and Perilipin 1 in transplanted beige adipocytes in recipient mice with or without doxycycline solution gavage. Scale bar = 100 μM. **(D)** Body weight of the mice. n = 4 Sham + Veh and Sham + Dox, n = 8 Beige + Veh and Beige + Dox. **(E)-(I)** Metabolic rate of the mice was measured by indirect calorimetry. **(E)** Oxygen consumption (VO_2_) in light and dark cycles of the mice. **(F)** VO_2_ rhythms in mice receiving beige cell engraftment. **(G)** Energy expenditure in light and dark cycles of the mice. **(H)** Energy expenditure rhythms in mice receiving beige cell engraftment.** (I)** Energy expenditure of mice analyzed with ANCOVA. **(J)** GTT of the mice. **(K)** The area under the curve (AUC) in** (J)**. All data are presented as mean with SD. Statistical significance was calculated by unpaired two-tailed Student's *t* test [**(D)** to **(H)**, **(J)**], one way ANOVA **(K)** or ANCOVA **(I)** (*P < 0.05).

As LETMD1 enhanced energy metabolism in human beige adipocytes *in vitro*, we next examined the effect of LETMD1 in human beige adipocytes *in vivo*. To this end, hiPSCs, with an inducible overexpression of *LETMD1* by doxycycline, were differentiated into beige adipocytes *in vitro*, followed by transplantation into the hindlimb muscle of the NOD/SCID mice 24. Transplantation at the hindlimb muscle, on one hand, better assists the survival of the transplanted beige adipocytes, and on the other hand, facilitates a more distinct and accurate evaluation of the successful transplantation of the beige adipocytes. The expression of *LETMD1* in the transplanted beige adipocytes was stimulated by oral supplementation of doxycycline solution (10 mg/ml) daily for five days (Figure 4A). H&E staining of the muscle sections at the injection site confirmed the successful transplantation of human beige adipocytes (Figure 4B). Furthermore, doxycycline-inducible *LETMD1* expression in transplanted human beige adipocytes was visualized by immunofluorescence staining (Figure 4C). Functionally, compared to those receiving sham operation plus treatment with vehicle (Sham + Veh), mice engrafted with human beige adipocytes (Beige-Veh) showed a modest reduction in body weight (Figure 4D). More importantly, compared to the Beige-Veh group, supplementation of doxycycline to beige cell transplanted mice (Beige + Dox) more efficiently reduced body weight (Figure 4D) and increased whole body energy expenditure (Figure 4E-I). In contrast, no significant difference in body mass and energy expenditure was detected between the Sham-Veh and Sham-Dox groups (Figure 4D-I). GTT demonstrated that transplantation of human beige adipocytes improved glucose utilization in recipient mice (Beige + Veh vs. Sham + Veh), and this beige adipocyte transplantation-conferred effect was further potentiated after doxycycline-induced overexpression of *LETMD1* (Beige + Dox vs. Beige + Veh) (Figures 4J-K).

**Figure 5:**
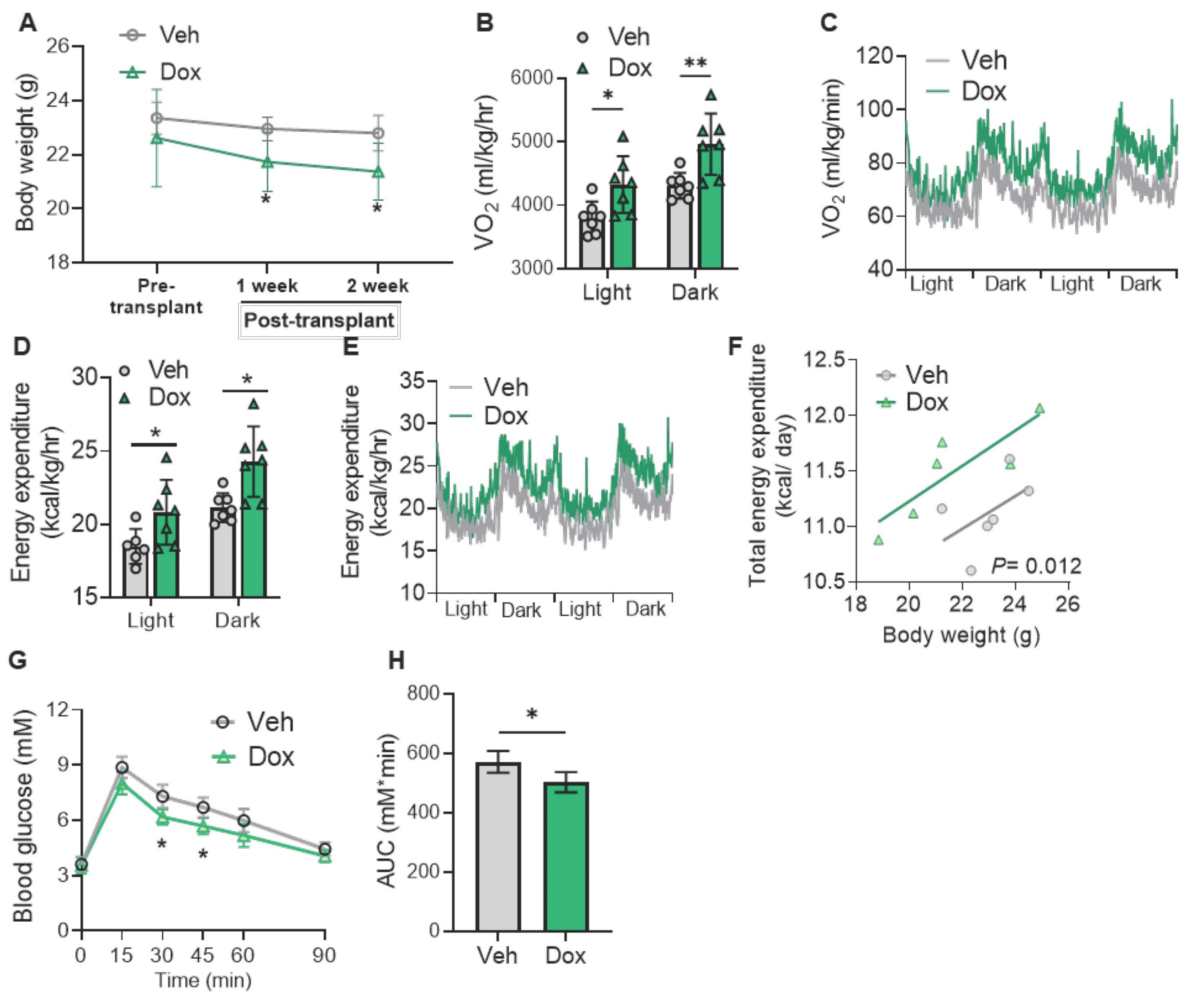
** LETMD1 induces UCP1-independent energy expenditure in human beige adipocytes.** *UCP1* KO hiPSC line with doxycycline-inducible *LETDM1* expression was differentiated *in vitro* to beige adipocytes and transplanted to NOD/SCID mice as in Figure 4. After recovery, the mice were orally gavaged with doxycycline (Dox) or vehicle (Veh) for two weeks. **(A)** Body weight of the mice. n = 6 for each group. **(B)-(F)** Metabolic rate of the mice was measured by indirect calorimetry. n = 6 for each group.** (B)** VO_2_ in light and dark cycles of the mice. **(C)** VO_2_ rhythms in mice receiving beige engraftment. **(D)** Energy expenditure in light and dark cycles of the mice. **(E)** Energy expenditure rhythms in mice receiving beige engraftment. **(F)** Energy expenditure of mice analyzed with ANCOVA. **(G)** GTT of the mice.** (H)** The AUC in **(G)**. n = 6 for each group. All data are presented as mean with SD. Statistical significance was calculated by unpaired two-tailed Student's *t* test [**(A)**, **(B)**, **(D)**,** (G)**, **(H)**] or ANCOVA **(F)** (*P < 0.05, **P < 0.01, and ***P < 0.001).

Furthermore, when *UCP1* KO hiPSCs with the inducible* LETMD1* expressing cassette were differentiated into beige adipocytes and transplanted into the mice in a similar strategy, upon doxycycline supplementation, a significant reduction in body weight was observed in mice receiving the *UCP1* KO-*LETMD1* overexpression beige adipocytes (Figure 5A). Likewise, compared with the vehicle group, mice fed with doxycycline to induce *LETMD1* in *UCP1* KO beige adipocytes had significantly increased VO_2_ (Figure 5B-C), energy expenditure (Figures 5D-F) and improved glucose intolerance (Figures 5G-H). These results demonstrated that LETMD1 action occurs through a UCP1-independent mechanism *in vivo*.

### Establishment of LETMD1 reporter hiPS cell line

**Figure 6:**
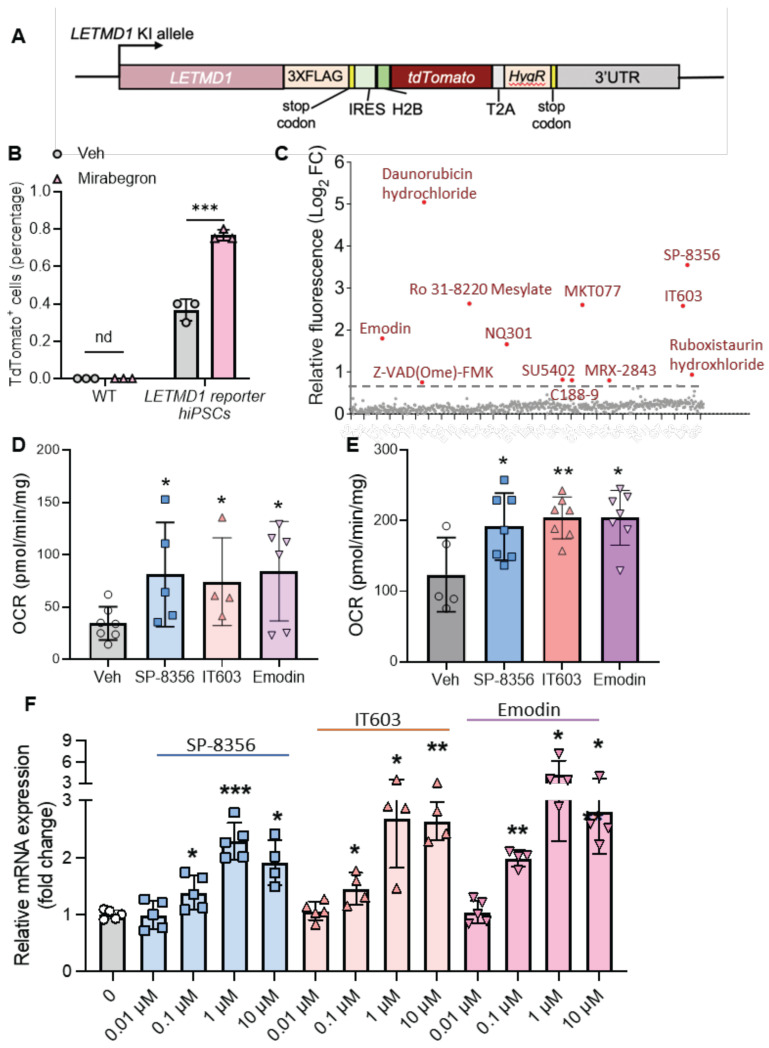
** Screening of pre-clinical compounds enhancing *LETMD1* expression. (A)** Illustration of the knock-in allele of the *LETMD1-tdTomato* reporter hiPSC. **(B)** WT and *LETMD1* reporter hiPSCs were treated with β3 adrenergic receptor agonist Mirabegron for 24 h. Relative fluorescence intensity of the cells was measured. **(C)-(F)** *LETMD1-tdTomato* reporter hiPSCs were differentiated to beige adipocytes. A pre-clinical compound library comprising 709 compounds were used to treat the adipocytes.** (C)** Scatter plot displaying relative fluorescence intensity of individual compounds (10 μM, 24 h). Compounds showing >1.5 fold increase of relative fluorescence intensity are highlighted in red. Results are the average of three independent experiments.** (D)-(E)** Functional validation of the compounds. WT **(D)** and *UCP1* KO **(E)** hiPSC-derived beige adipocytes were treated with the SP-8356, IT603, and Emodin (1µM) for 24 h. DMSO was used as the vehicle control. Basal OCR of the beige adipocytes was measured by Seahorse bioanalyzer. n = 7 for vehicle, n = 5 for SP-8356, n = 4 for IT603, n = 6 for Emodin. **(F)** Validation of the compounds at the mRNA level. *LETMD1-tdTomato* reporter hiPSCs were differentiated to beige adipocytes followed by treatment with the candidate compounds at various concentrations for 24 h. mRNA levels of *LETMD1* were examined by realtime PCR. n = 4-5. DMSO was used as the vehicle control. All data are presented as mean with SD. Statistical significance was calculated by unpaired two-tailed Student's *t* test** (B)** or one way ANOVA **(D-F)** (*P < 0.05, **P < 0.01, and ***P < 0.001).

Our findings suggest LETMD1 is a valid target to increase energy expenditure in human beige adipocytes. Although β3 adrenergic receptor agonists are known to increase *LETMD1* expression, the existence of safe and efficient pharmacological reagents enhancing *LETMD1* expression in human beige adipocytes are currently unavailable. In order to achieve that, a fluorescent protein-based *LETMD1* reporter hiPS cell line was generated (Figure 6A and S5A). Using the homology-directed repair (HDR), the fluorescent reporter, *TdTomato* gene, was inserted before the stop codon of the endogenous *LETMD1* gene linked with a 2A peptide; this arrangement enables measurement of TdTomato fluorescence with signal quantitation that is proportional to endogenous *LETMD1* expression (Figure S5A).

The reporter hiPSC was verified by DNA sequencing (Figure S5B) and showed similar capacity of beige adipocyte differentiation to the WT hiPSC (Figure S5C). The β3 adrenergic receptor agonist, Mirabegron, was used as a positive control to confirm the concomitant TdTomato fluorescence intensity and endogenous *LETMD1* expression, since Mirabegron readily increased *LETMD1* mRNA expression in human beige adipocytes (Figure S5D). Flow cytometry analysis showed a significant increase in the number of tdTomato-positive cells compared to those treated with the vehicle solution (Figure 6B). This finding was consistent with the microscopy imaging results that the TdTomato fluorescence intensity of the beige adipocytes was increased after treatment of Mirabegron (Figure S5E). These results collectively indicated that our reporter cell line is correctly established and faithfully reports the expression level of the endogenous *LETMD1* gene.

### Screening of compounds that elevate LETMD1 expression

Using this *LETMD1* reporter hiPSC, a preclinical compound library which includes 709 preclinical small molecule compounds with well-defined targets, was screened for their potential to enhance *LETMD1* expression. They are structurally diverse, potent, and cell-penetrable, and can be used in high-throughput and high-intensity screening. *LETMD1* reporter hiPSCs were differentiated to beige adipocytes, followed by incubation of each compound for 24 h before measurement of TdTomato fluorescence intensity. The experiment was repeated three times and eventually 12 compound hits that consistently elevated the TdTomato fluorescence intensity were identified (Figure 6C and Supplementary Table 2). Fluorescence imaging of the treated beige adipocytes for DAPI and spontaneous red fluorescence also showed that these 12 compounds identified significantly increased the level of TdTomato fluorescence signal in the reporter beige adipocytes compared to the vehicle (Figure S5F).

Next, we functionally examined the effect of these 12 candidate compounds on WT and *UCP1* KO human beige adipocytes. Three of these compounds, SP-8356, IT603 and Emodin, significantly and consistently increased the OCR in both WT and *UCP1* KO human beige adipocytes (Figure 6D-E). In addition, these three compounds induced the mRNA levels of *LETMD1* in beige adipocytes in a dose-dependent manner (Figure 6F).

**Figure 7:**
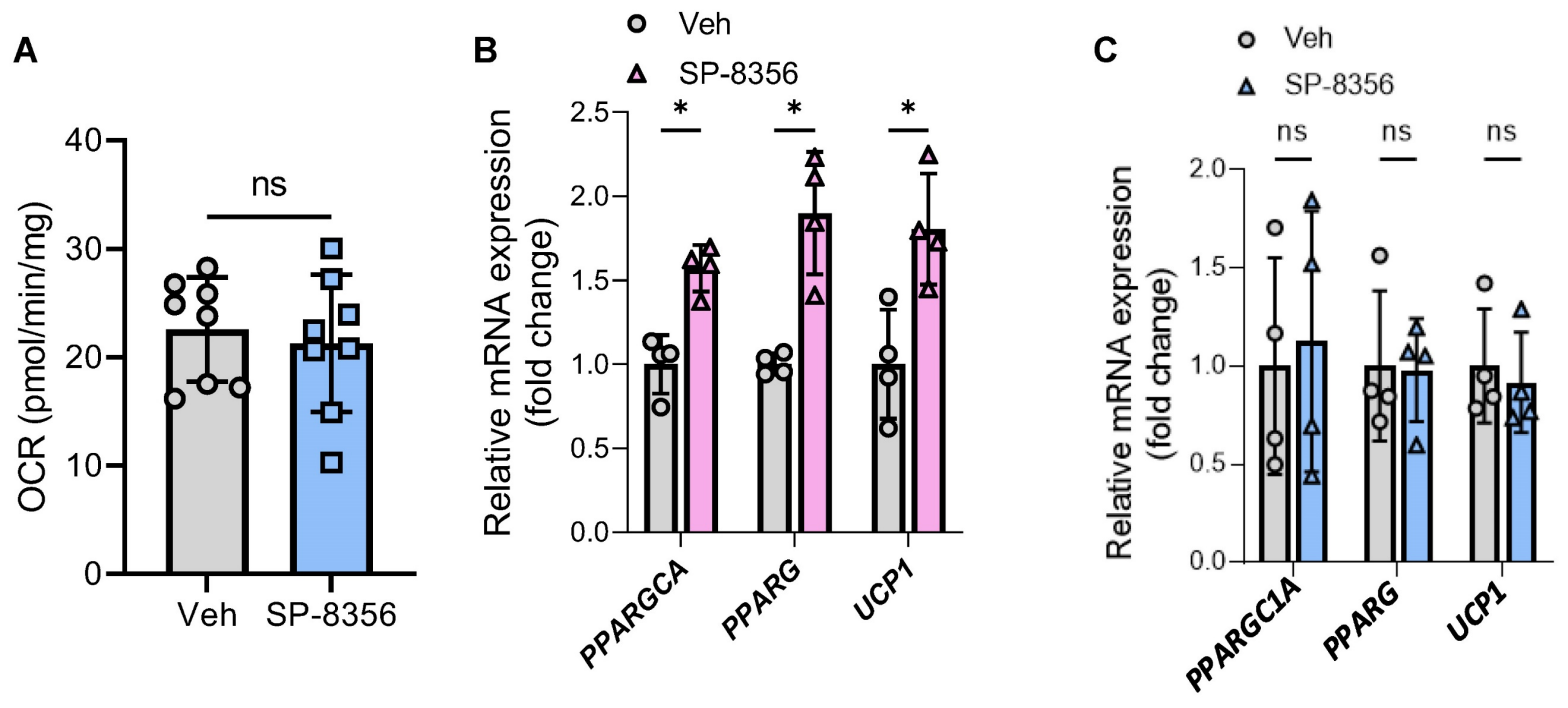
** SP-8356 enhances beige adipocyte energy metabolism via LETMD1.** HiPSCs were differentiated to beige adipocytes followed by treatment with SP-8356 (1µM) for 24 h. DMSO was used as the vehicle control. OCR was measured by Seahorse bioanalyzer. **(A)** Basal respiration rate in *LETMD1* KO hiPSC-derived beige adipocytes. n = 8 for each group. **(B)** Relative mRNA expression of *PPARGC1A, PPARG,* and* UCP1* in WT hiPSC-derived beige adipocytes treated with or without SP-8356. n = 4 for each group. **(C)** Relative mRNA expression of *PPARGC1A, PPARG,* and* UCP1* in *LETMD1* KO hiPSC-derived beige adipocytes treated with or without SP-8356. n = 4 for each group. All data are presented as mean with SD. Statistical significance was calculated by unpaired two-tailed Student's *t* test (*P < 0.05).

Among these three compounds, SP-8356 showed the highest therapeutic potential since it is an orally active drug 25. Furthermore, the enhancement effect of SP-8356 on human beige adipocyte oxygen consumption was completely lost in *LETMD1* KO human beige adipocytes (Figure 7A), indicating that the action of SP-8356 was dependent on LETMD1. Accordingly, while SP-8356 increased the mRNA level of beige adipocyte marker genes in WT beige adipocytes (Figure 7B), such effects were abolished in *LETMD1* KO beige adipocytes (Figure 7C). SP-8356 is a potent antagonist of cluster of differentiation 147 (CD147). *CD147* mRNA expression was readily detected in human beige adipocytes, especially after differentiation (Figure S6).

### SP-8356 protects against diet-induced obesity and metabolic disorders

**Figure 8:**
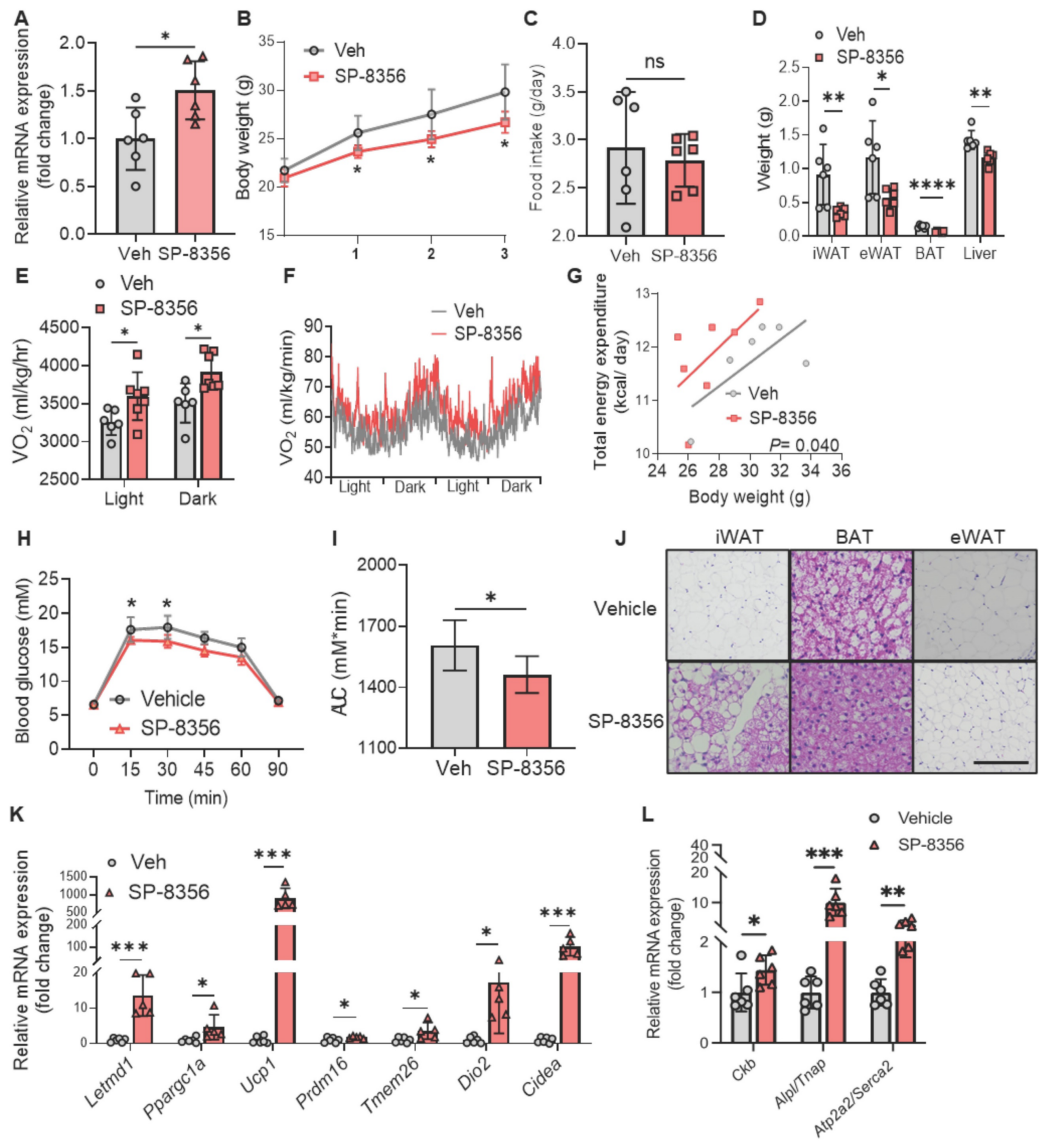
** SP-8356 improves energy expenditure and protects against HFD-induced obesity and glucose intolerance in mice. (A)** SVCs from *Ucp1* KO mice were differentiated to beige adipocytes, followed by incubation with vehicle (Veh) or SP-8356 (1µM) for 24 h. The relative mRNA level of *Letmd1* was examined. n = 6. **(B)-(L)** C57BL/6J male mice were fed with vehicle (Veh) or SP-8356 while fed with HFD for 3 weeks. **(B)** Body weight, **(C)** Food intake, and **(D)** Tissue weight of the mice. n=6. **(E)-(G)** Metabolic rate of the mice was monitored by indirect calorimetry. **(E)** Average O_2_ consumption (VO2). n = 6 for vehicle, n = 7 for SP-8356. **(F)** VO_2_ rhythms in mice. n = 6 for vehicle, n = 7 for SP-8356. **(G)** ANCOVA of energy expenditure in mice. n = 6 for vehicle, n = 7 for SP-8356. **(H)** GTT of the mice. n = 6 for each group. **(I)** AUC in **(H)**. **(J)** H&E staining of the mice adipose tissues. Scale bar = 100 μM. **(K)** Relative mRNA expression of classical beige marker genes in iWAT. n = 6 for vehicle, n = 5 for SP-8356. **(L)** Relative mRNA expression of UCP1-independent thermogenic genes in iWAT. n = 6 for each group. All data are presented as mean with SD. Statistical significance was calculated by unpaired two-tailed Student's *t* test or ANCOVA **(G)** (*P < 0.05, **P < 0.01, ***P < 0.001).

We proceeded to evaluate whether SP-8356 boosts metabolism in animal models. We first confirmed that SP-8356 increased LETMD1 expression in mice beige adipocytes. The stromal vascular cells (SVC) from *Ucp1* KO mice were isolated and differentiated to beige adipocytes *in vitro*, followed by treatment with 1 μM of SP-8356 for 24 h. SP-8356 significantly increased the mRNA level of *Letmd1* in mice beige adipocytes (Figure 8A). This finding suggests that the pathway mediating SP-8356-elicited LETMD1 expression in beige adipocytes is retained in mice.

Next we investigated whether SP-8356 exerts a positive effect on metabolic function in mice. Male C57BL/6J mice were randomly divided into two groups, and the mice were fed with either vehicle or SP-8356 (50 mg/kg/d) respectively together with HFD for 3 weeks (Figure S7A). Compared to those fed with the vehicle solution, there was a significant reduction in body weight in mice after supplementation of SP-8356 (Figure 8B), while no significant difference in food intake was observed between these two groups (Figure 8C). The weight of adipose tissues, including the white and brown adipose tissues, was substantially reduced in SP-8356-treated mice (Figure 8D). These results demonstrated that SP-8356 effectively antagonizes diet-induced obesity in animal model.

Furthermore, whole body oxygen consumption and energy expenditure were remarkably elevated in obese mice after treatment of SP-8356 (Figures 8E-G). We also observed a significant improvement in glucose intolerance in SP-8356-treatment group compared to the vehicle control group (Figure 8H-I). Similarly, when SP-8356 was fed to mice with STC diet, a similar but more modest change in reducing adiposity, and an increased energy expenditure was observed (Figure S8A-I). Although no significant change in body weight was observed, the mice exhibited an improved glucose clearance rate after SP-8356 treatment (Figure S8J-K). This is consistent with the current understanding that beige adipocytes might confer metabolic protection independent of body weight reduction 5, 6.

Histological analysis showed that mice receiving SP-8356 treatment had more beige adipocytes in their subcutaneous iWAT as evidenced by the presence of multilocular cells (Figure 8J). The lipid droplet size in BAT and eWAT was also much smaller (Figure 8J). More importantly, the expression level of *Letmd1* was increased by over 10 fold in mice iWAT following SP-8356 treatment (Figure 8K). In addition, the expression of other beige adipocyte marker genes, including *Ppargc1a*, *Ucp1*, *Prdm16*, *Tmem26*, *Dio2*, and *Cidea*, was markedly increased in iWAT following SP-8356 treatment, compared to the vehicle control (Figure 8K). Notably, consistent with our findings in human beige adipocytes, the expression of those genes, involved in UCP1-independent energy expenditure including not only *Ckb, Alpl,* but also *Atp2a2*, was significantly upregulated in iWAT by SP-8356 (Figure 8L). In contrast, the effect of SP-8356 on BAT was modest (Figure S7B), suggesting that the effect of SP-8356 is more specific to beige adipocytes.

## Discussion

LETMD1 is a newly identified key regulator in the maintenance and activation of brown and beige adipocytes in preclinical models 14-16, 18, 26. Our study is the first to investigate the function of LETMD1 in the context of human beige adipocytes. We provided both *in vitro* and *in vivo* evidence that enhancing *LETMD1* expression in human beige adipocytes potentiates energy expenditure *in vitro* and in mice engrafted of the human beige adipocytes. The increased energy expenditure is accompanied by an improvement of glucose intolerance, highlighting the potential of LETMD1 as a new therapeutic target for obesity and metabolic diseases.

Activation of beige adipocytes and thermogenesis is important for maintaining energy balance and metabolic health 27. Mitochondrial biogenesis is a key event in activation of beige adipocytes. LETMD1 has previously been reported by several groups to play critical roles in BAT with multiple mechanisms of action 14-16, 18, 26. Although first reported as a mitochondrial outer membrane protein 13, Choi *et al.* reported that LETMD1 is also present in the nucleus of brown adipocytes and regulates the transcription of thermogenic genes via interacting with BRG1 18. Consistent with this finding, LETMD1 is also detected within the cell nucleus of human beige adipocytes, suggesting that the function of LETMD1 to enhance energy expenditure in human beige adipocytes is at least partially mediated through its action on directly modulating gene transcription. It is worth noting that *PPARGC1A* is upregulated upon overexpression of *LETMD1*, and that BRG1 binding on the *Ppargc1a* gene locus is mitigated in *Letmd1* KO adipocytes, suggesting that the transcription of *PPARGC1A* is directly regulated by the nuclear-resident LETMD1. PGC1α is well known as the master regulator of mitochondrial biogenesis and function 21. Mechanistically, PGC1α induces the expression of *NRF1* and* NFE2L2*, followed by enhanced transcription of *TFAM*. NRF1, NFE2L2 and TFAM are key transcription factors facilitating mitochondrial biogenesis / function 22. We found that the expression of these three key genes downstream of PGC1α was significantly increased upon overexpression of *LETMD1*, highlighting the hypothesis that LETMD1 facilitates mitochondrial biogenesis and function in human beige adipocytes via boosting the transcription of *PPARGC1A.*

There is an emerging realization that in human beige adipocytes, UCP1-independent heat generation, which mainly relies on engaging futile substrate cycles to accelerate ATP consumption, contributes significantly to whole body energy consumption 28. This conclusion is consistent with sn-Seq-based analysis in recent years where UCP1+ adipocytes are barely detected in human WAT 12. Mechanistically the creatine and intracellular calcium cyclings, both of which consume large amounts of ATP, are reported as alternative mechanisms of adaptive thermogenesis in beige adipocytes 5, 6. Interestingly, ATP5K+ beige adipocytes present in both mice and human adipose tissue engage in UCP1-independent energy expenditure via creatine futile cycling 7, 8. However, the biogenesis of these non-canonical beige adipocytes remains unclear. Interestingly, overexpression of *LETMD1* increased the expression of a number of genes encoding the ATP5 subunits in human beige adipocytes, together with significantly elevated expression of genes in creatine futile cycling. This finding suggests that LETMD1 is an upstream activator of ATP5K+ beige adipocytes. By comparison, genes involved in calcium futile cycling, which happens between ER and cytosol, were not altered after *LETMD1* overexpression in human beige adipocytes, further highlighting the specific action of LETMD1 in mitochondrial remodeling. This hypothesis is corroborated by our finding that abrogation of creatine futile cycling largely reversed LETMD1 overexpression-evoked OCR in human beige adipocytes.

Currently, safe and efficient pharmacological strategies to stimulate beige adipocyte activity, especially the UCP1-independent branch, are unavailable 29. Considering the translational value of LETMD1 in enhancing beige adipocyte activity, a high-throughput screening platform for *LETMD1* expression was established and applied to search for small molecules that enhance *LETMD1* expression in human beige adipocytes. SP-8356 was identified as a strong hit showing robust efficacy both *in vitro* and *in vivo*. SP-8356 is an anti-inflammatory synthetic verbenone derivative and first reported to inhibit breast cancer progression 30. It was later identified as an antagonist to CD147 20. CD147 is a type I transmembrane glycoprotein of the immunoglobulin superfamily broadly expressed on the surface of various cell types. *In vivo* studies showed that SP-8356 reduces mitochondrial membrane potential (MMP) activity by inhibiting the dimerization of CD147, and combats vascular diseases, including neointimal hyperplasia and arterial stiffness 20, and atherosclerosis 25.

Here we add to the health benefits of SP-8356 to anti-obese and anti-metabolic diseases. Intriguingly, the impact of SP-8356 to stimulate beige adipocyte activity was solely mediated by LETMD1 since the compound completely lost its effect in *LETMD1* KO beige adipocytes. The mechanism by which SP-8356 stimulates *LETMD1* expression is not completely understood. However SP-8356 reportedly suppresses nuclear factor kappa-B (NF-κB) signaling in breast cancer cells 30. In line with this finding, several compound hits in our high-throughput screening are inhibitors to NF-κB signaling pathway, suggesting the possibility that SP-8356 also enhances *LETMD1* expression through inhibition of NF-κB signaling.

## Conclusions

In summary, the current study demonstrates LETMD1 as a pivotal player in enhancing non-canonical, UCP1-independent energy expenditure in human beige adipocytes, and therefore establishes it as a druggable target for obesity and metabolic diseases. SP-8356, a previously known anti-inflammation small compound, shows promising potential as a new drug lead to combat obesity and metabolic diseases via targeting LETMD1.

### Limitations of the study

We cannot exclude the possibility that LETMD1 also directly regulates the function of mitochondria via other mechanisms. The detailed molecular events downstream of CD147 to turn on the expression of *LETMD1*, upon stimulation of SP-8356 in beige adipocytes, also remains to be investigated in future.

## Supplementary Material

Supplementary figures and tables 1-2.

Supplementary table 3.

## Abbreviations

AEEC: Animal Experimentation Ethics Committee
ANCOVA: analysis of covariance
AUC: area under the curve
BAT: brown adipose tissue
CD147: cluster of differentiation 147
cDNA: complementary DNA
DEG: differentially expressed gene
DMEM: Dulbecco's Modified Eagle Medium
FBS: fetal bovine serum
GTT: glucose tolerance test
HFD: high fat diet
hiPSC: human induced pluripotent stem cell
IBMX: isobutylmethylxanthine
KO: knockout
LETMD1: leucine zipper-, EF-hand-containing transmembrane protein 1 domain containing 1
OCR: oxygen consumption rate
PGC1α: peroxisome proliferator-activated receptor-γ coactivator-1α
sgRNA: single guide RNA
STC: standard chow
SVC: stromal vascular cell
UCP1: uncoupling protein 1
WAT: white adipose tissue
WT: wildtype
