# Supplementary material for: LETM-domain containing 1 (LETMD1) protects against obesity via enhancing UCP1-independent energy expenditure in human beige adipocytes
Source: Theranostics. 2025 Jan 2;15(5):1914–29. doi: 10.7150/thno.104568 (PMC11780525; doi:10.7150/thno.104568)
Supplement: Supplementary file 1 — Supplementary figures and tables 1-2. [file thnov15p1914s1.pdf]

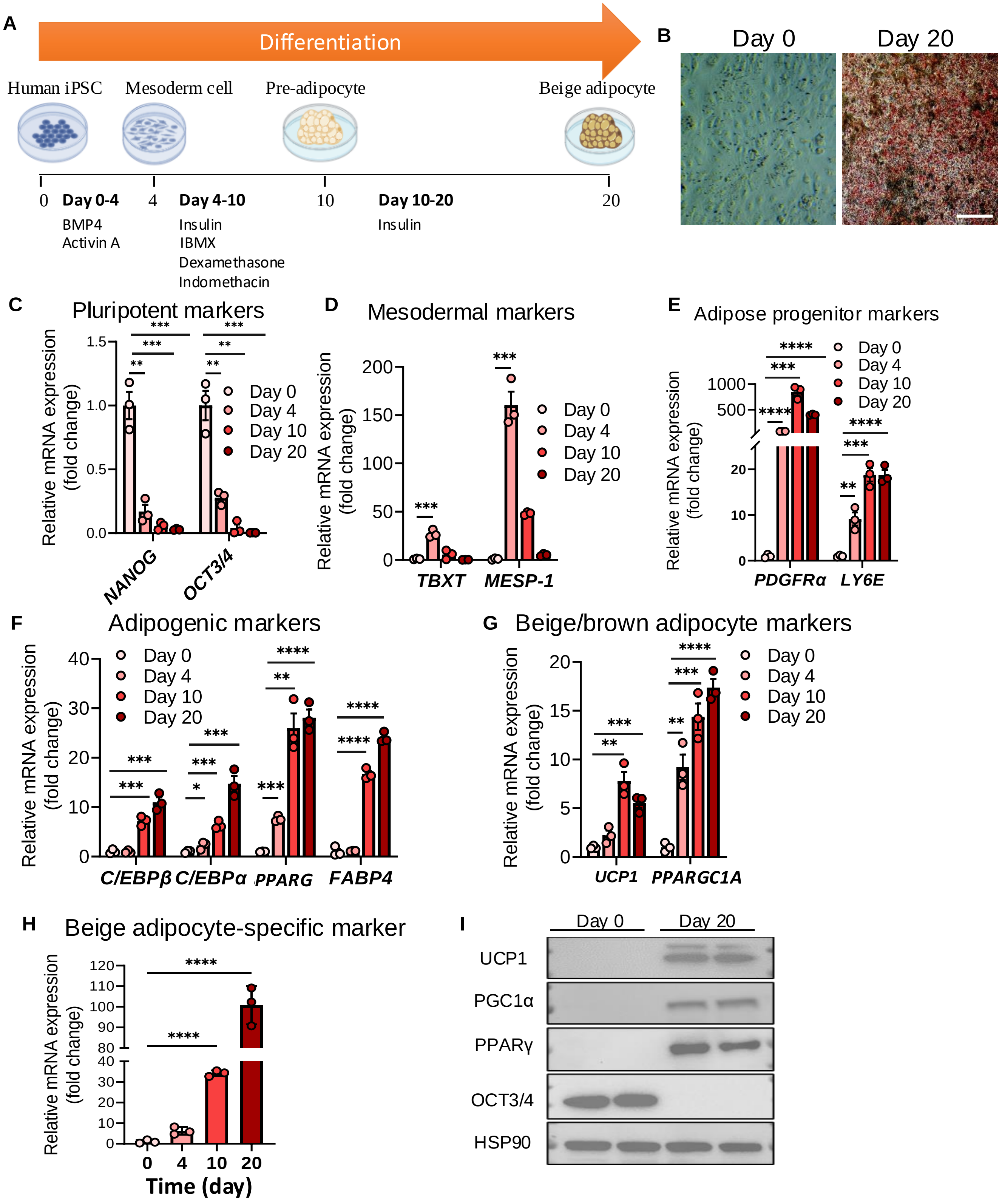

Figure S1. Differentiation of human beige adipocyte from hiPSC.
(A) The experimental procedures for the differentiation of hiPSCs into beige adipocytes. (B) Oil red O (ORO) staining of adipocyte-differentiated hiPSCs on day 0 and day 20. Scale bar = 100 μM. (C)-(H) Time-course mRNA expression of indicated pluripotent markers, mesodermal transcription factors, adipose progenitor markers, adipogenic transcriptional markers, beige/brown adipocyte markers, and the beige adipocyte-specific marker. n = 3 for each group. (I) Western blot analysis of protein expression of UCP1, PGC1α, PPARγ, OCT3/4, and HSP90 (loading control) on day 0 and day 20. All data are presented as mean with SD. Statistical significance was calculated by one way ANOVA (**P* < 0.05, ***P* < 0.01, ****P* < 0.001, and *****P* < 0.0001).

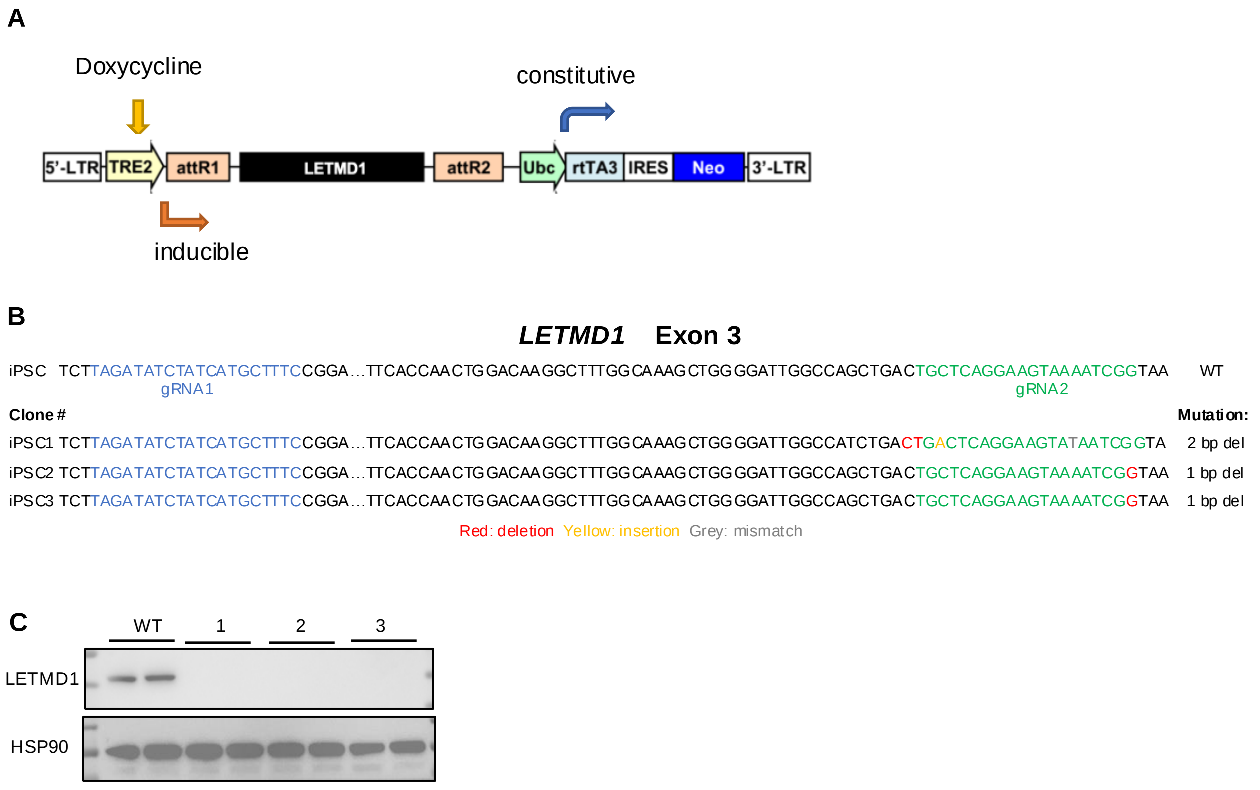

Figure S2. Generation of *LETMD1* overexpressing and knockout hiPSCs.
(A) Schematic illustration of the DNA construct harboring the Dox-inducible *LETMD1* cassette. pINDUCER is the vector backbone. (B)-(C) Generation of *LETMD1* KO hiPSC. *LETMD1* gene in hiPSC was deleted by CRISPR-Cas9 mediated strategy. (B) Genomic sequences of CRISPR-Cas9-edited hiPS single-cell clones 1, 2, and 3 demonstrate on-target deletion mutations in the *LETMD1* gene. Genetic alterations for every single clone were confirmed by Sanger DNA sequencing. (C) Western blot analysis of protein expression of LETMD1 and HSP90 (loading control) in WT and *LETMD1* KO hiPS single-cell clone-derived beige adipocytes.

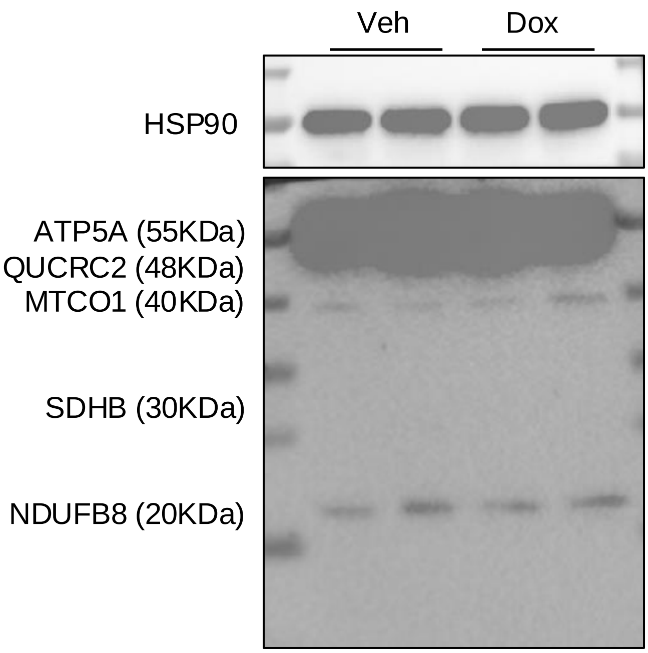

Figure S3. LETMD1 does not alter mitochondria complex expression.
Doxycycline-inducible *LETMD1* hiPSCs were differentiated into beige adipocytes and treated with doxycycline (Dox) or vehicle (Veh) solution for 24 h before analysis. Western blot analysis of protein expression of OXPHOS in human beige adipocytes.

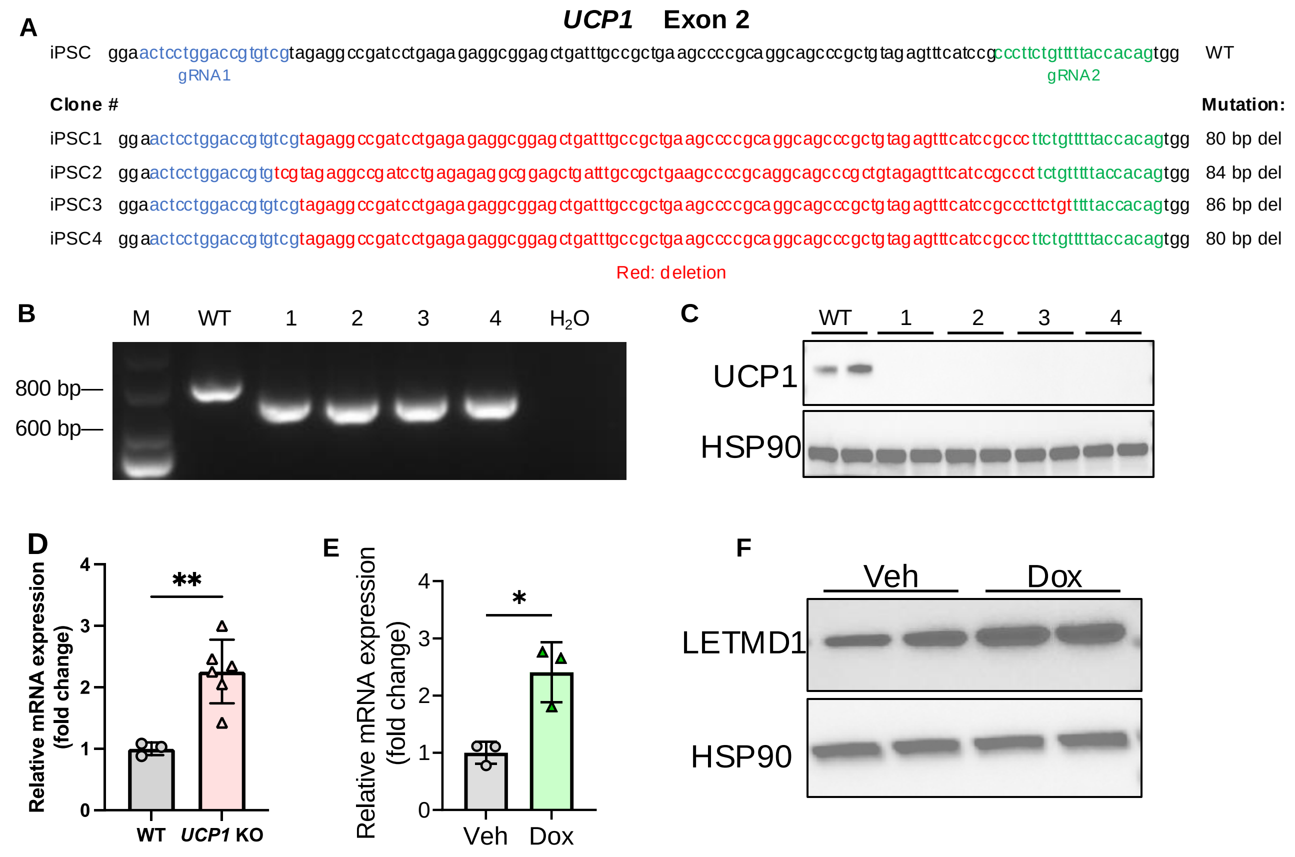

Figure S4. Generation of *LETMD1* inducible hiPSCs in *UCP1* knockout background.
(A) Genomic sequences of CRISPR-Cas9-edited hiPS single-cell clones 1, 2, 3, and 4 demonstrate on-target deletion mutations in the *UCP1* gene. Genetic alterations for every single clone were confirmed by Sanger DNA sequencing. (B) Representative PCR screening results in *UCP1* KO hiPS single-cell clones. (C) Western blot analysis of protein expression of UCP1 in WT and *UCP1* KO hiPS single-cell clone-derived beige adipocytes. (D) *LETMD1* mRNA expression in WT and *UCP1* KO beige adipocytes. n = 3 for WT, n = 6 for *UCP1* KO cells. (E) Relative mRNA expression of *LETMD1* gene in *UCP1* KO beige adipocytes. n = 3 for each group. (F) Western blot analysis of protein expression of LETMD1 in *UCP1* KO beige adipocytes. All data are presented as mean with SD. Statistical significance was calculated by unpaired two-tailed Student's *t* test (**P* < 0.05, ***P* < 0.01,).

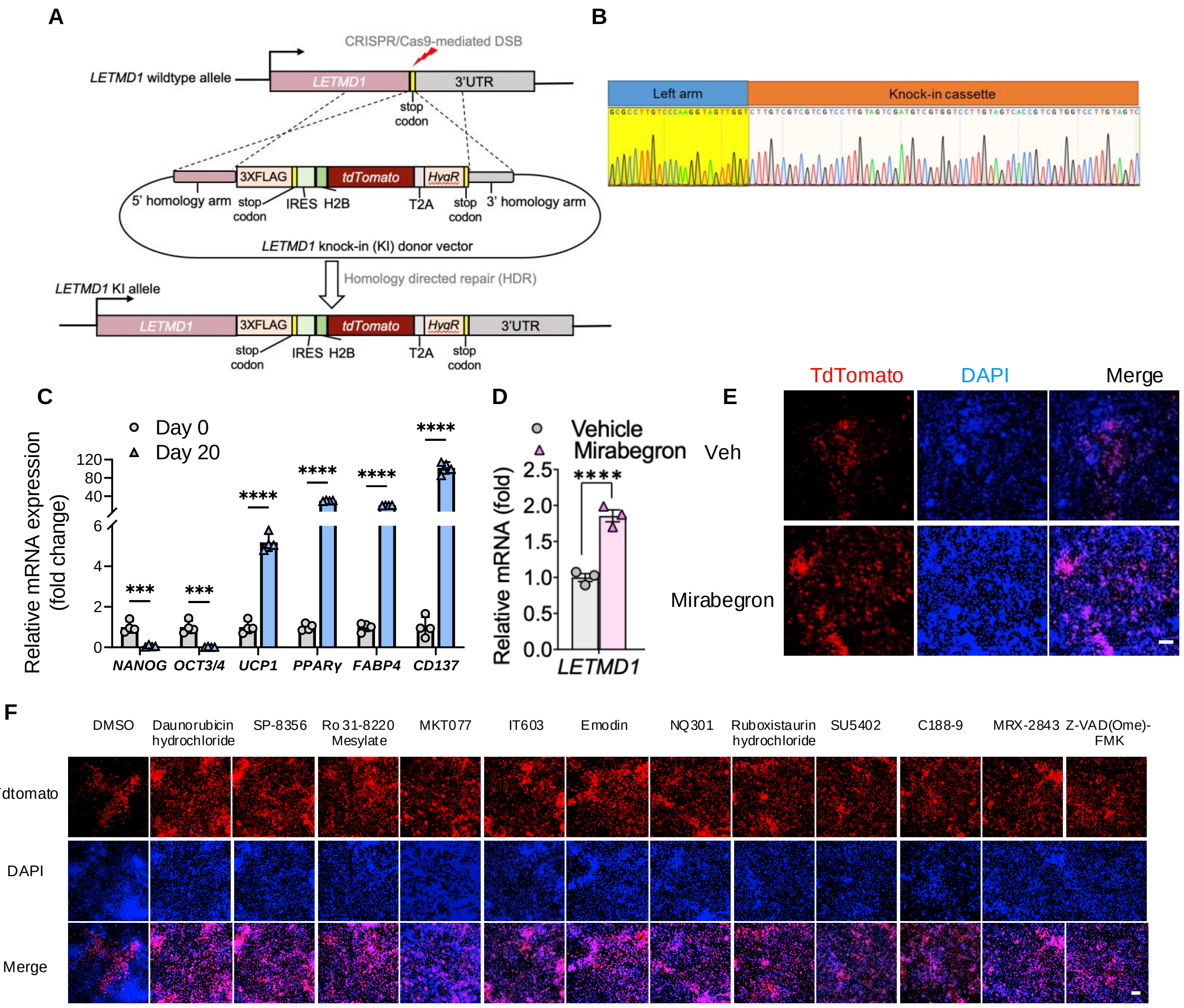

Figure S5. Generation and characterization of the *LETMD1-tdTomato* reporter hiPSCs using the CRISPR-Cas9 genome engineering.

(A) Schematic illustration of the knock-in design for the *LETMD1-tdTomato* reporter hiPSCs. (B) The gene sequence of the *LETMD1-tdTomato* reporter hiPSC was confirmed by Sanger DNA sequencing. (C) Relative mRNA expression of *NANOG*, *OCT3/4*, *UCP1*, *PPARγ*, *FABP4*, and *CD137* genes before and after differentiation of *LETMD1* reporter hiPSC. n = 4 for each group. (D) WT hiPSC was differentiated into beige adipocytes and treated with β3 adrenergic receptor (β3-AR) agonist Mirabegron for 24 h. The fold change of *LETMD1* mRNA was examined. n = 3 for each group. (E) Representative images of tdTomato in *LETMD1* reporter hiPSCs-derived beige adipocytes treated with or without Mirabegron for 24 h. Scale bar = 100 μM. (F) Representative images of tdTomato in *LETMD1* reporter hiPSCs-derived beige adipocytes treated with vehicle or other compounds. Scale bar = 100 μM. All data are presented as mean with SD. Statistical significance was calculated by unpaired two-tailed Student's *t* test (****P* < 0.001, and *****P* < 0.0001).

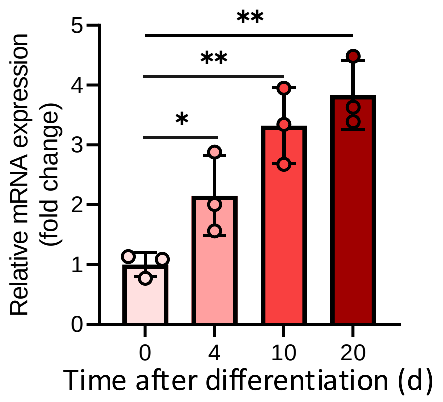

Figure S6. Expression of *CD147* gene during differentiation of hiPSCs.
The relative mRNA expression of *CD147* gene was examined during the differentiation of hiPSCs. n = 3 for each group. Data are presented as mean with SD. Statistical significance was calculated by one-way ANOVA (*$P < 0.05$, **$P < 0.01$).

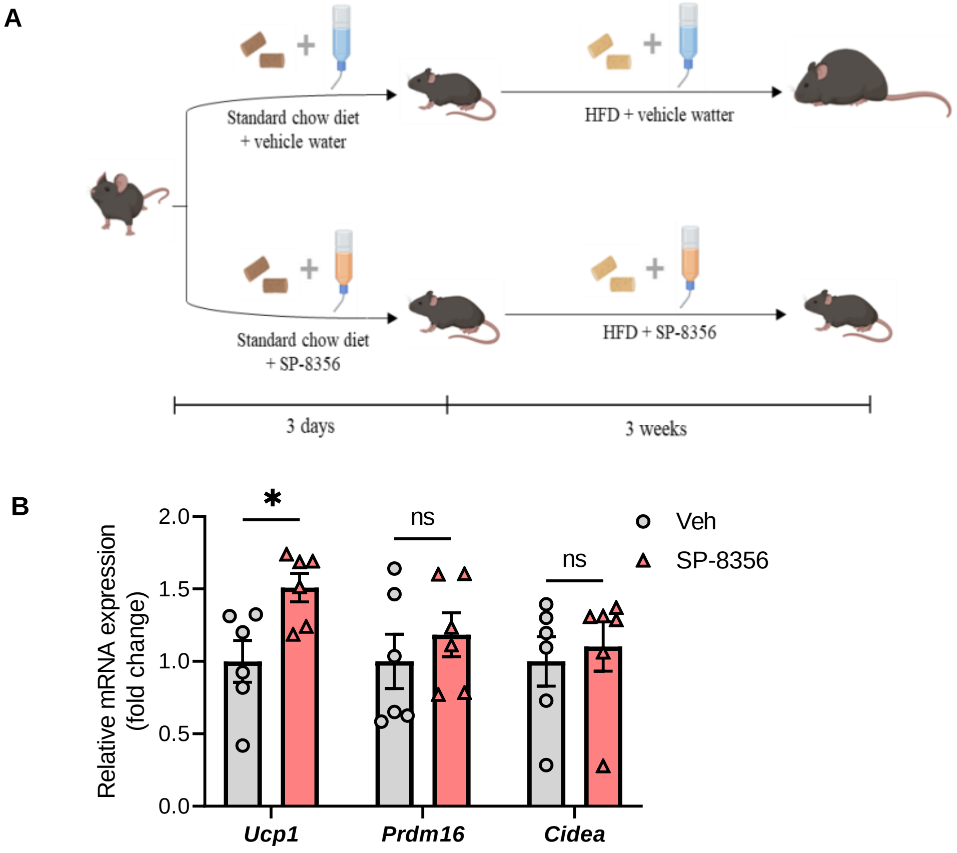

Figure S7. Mice were treated with SP-8356 while being fed a high fat diet.
(A) The experimental scheme of testing the effect of SP-8356 in vivo. (B) Relative mRNA expression of *Ucp1, Prdm16, and Cidea* genes in the BAT of the mice treated with or without SP-8356. n = 6 for each group. Statistical significance was calculated by unpaired two-tailed Student's *t* test (*$P < 0.05$).

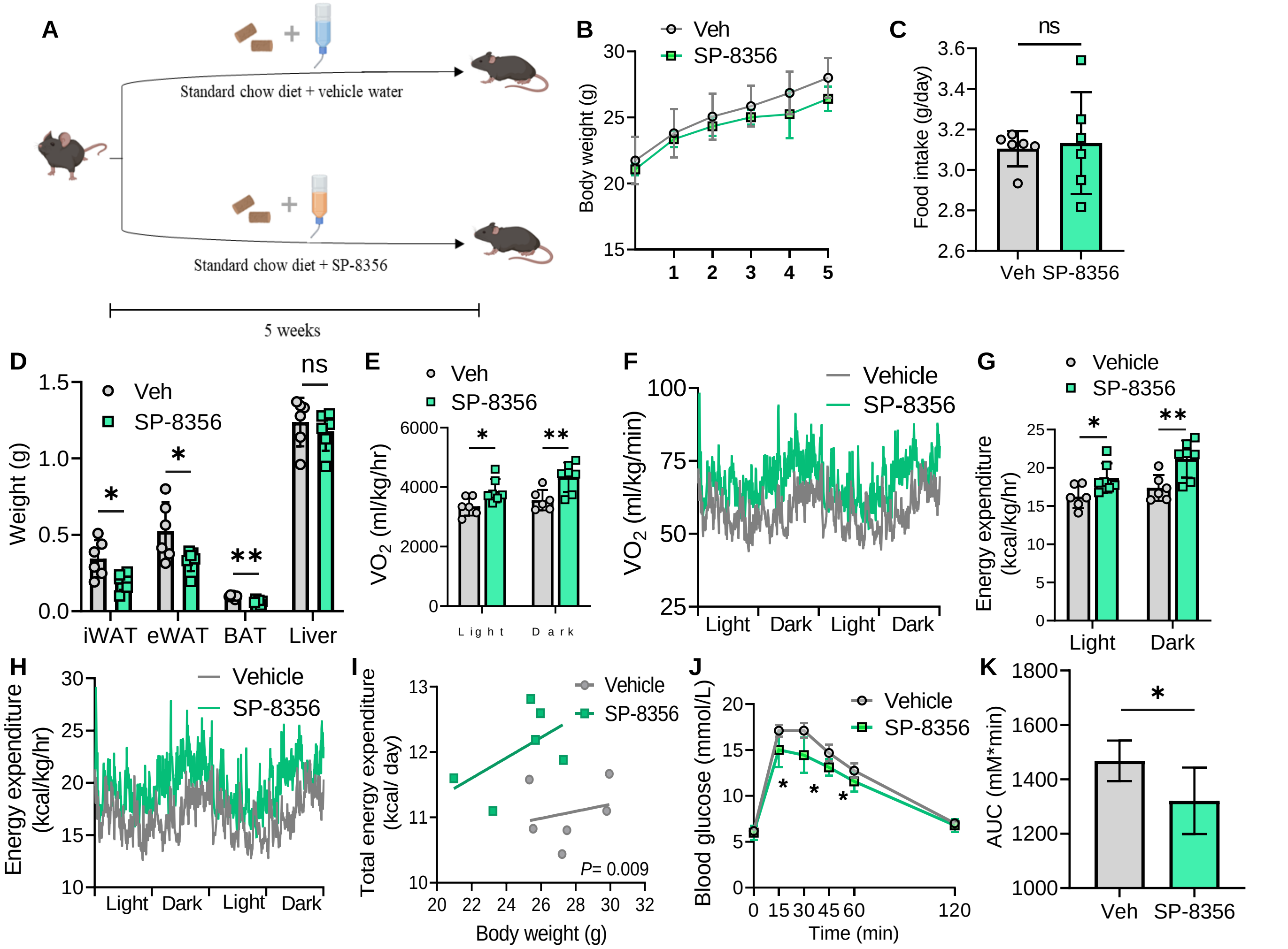

Figure S8. SP-8356 increases energy metabolism in standard chow diet (STC)-fed mice.
(A) The experimental scheme for treating STC-fed mice with SP-8356. (B) Body mass determination in STC-fed mice treated with or without SP-8356. n = 6 for each group. (C) Daily food intake calculation in STC-fed mice treated with or without SP-8356. n = 6 for each group. (D) Tissue weight of STC-fed mice treated with or without SP-8356. n=6 for each group. (E) STC-fed mice were assayed by indirect calorimetry. Average measurements of VO_2 for light and dark cycles are indicated. n = 6 for vehicle, n = 7 for SP-8356. Each dot represents one mouse. (F) VO_2 rhythms in STC-fed mice treated with or without SP-8356. n = 6 for vehicle, n = 7 for SP-8356. (G) Average measurements of energy expenditure for light and dark cycles from indirect calorimetry assays. n = 6 for vehicle, n = 7 for SP-8356. Each dot represents one mouse. (H) Energy expenditure rhythms in STC-fed mice treated with or without SP-8356. n = 6 for vehicle, n = 7 for SP-8356. (I) Total energy expenditure over 24 h versus body weight in STC-fed mice treated with or without SP-8356. n = 6 for each group. Each dot represents one mouse. The *p* value indicated is the ANOVA interaction effect. (J) GTT was performed in STC-fed mice. n = 6 for each group. (K) The AUC in (J). All data are presented as mean with SD. Statistical significance was calculated by unpaired two-tailed Student's *t* test or ANCOVA (I) (**P* < 0.05, ***P* < 0.01).

Gene name		Primer sequence
Human *β-actin*	Forward	5’-AGATCAAGATCATTGCTCCTCCTG-3’
	Reverse	5’-CAAGAAAGGGTGTAACGCAACTAAG-3’
Human *LETMD1*	Forward	5’-GTGGTAACCAAGACAAAAGCGA-3’
	Reverse	5’-GCATCAGCCCATAACATCTGC-3’
Human *NADH dehydrogenase 1*	Forward	5’-TGGCTCCTTTAACCTCTCCA-3’
	Reverse	5’-GGTTCGGTTGGTCTCTGCTA-3’
Human *Mt-CO2*	Forward	5’-CGATTGAAGCCCCCATTCGTA-3’
	Reverse	5’-CGATGGGCATGAAACTGTGGTT-3’
Human *NANOG*	Forward	5’-CTCACCGCAGGGAAAGAA-3’
	Reverse	5’-CAGGGCTGTCCTGAATAAGC-3’
Human *OCT3/4*	Forward	5’-CTCACCGCAGGGAAAGAA-3’
	Reverse	5’-AGGGTTTCTGCTTTGCAT-3’
Human *TBXT*	Forward	5’- GCTGTGACAGGTACCCAACC -3’
	Reverse	5’- CATGCAGGTGAGTTGTCAGAA -3’
Human *MESP-1*	Forward	5’- TCGAAGTGGTTCCTTGG -3’
	Reverse	5’- TGCTTGCCTCAAAGTGTC -3’
Human *PDGFRα*	Forward	5’- CTCACCGCAGGGAAAGAA -3’
	Reverse	5’- TCTTCAGGAAGTCCAGGTGAA -3’
Human *LY6E*	Forward	5’-GCCATCCTCTCCAGAATGAA-3’
	Reverse	5’-GCAGGAGAAGCACATCAGC-3’
Human *C/EBPβ*	Forward	5’-CTCACCGCAGGGAAAGAA-3’
	Reverse	5’-CCCTGCTCTGAGCTGTCG-3’
Human *C/EBPα*	Forward	5’-TGGACAAGAACAGCAACGAGTA-3’
	Reverse	5’-ATTGTCACTGGTCAGCTCCAG-3’
Human *PPARG*	Forward	5’-GGGATCAGCTCCGTGGATCT-3’
	Reverse	5’-TGCACTTTGGTACTCTTGAAGTT-3’
Human *FABP4*	Forward	5’-ACTGGGCCAGGAATTTGACG-3’
	Reverse	5’-CTCGTGGAAGTGACGCCTT-3’
Human *UCP1*	Forward	5’-CTCACCGCAGGGAAAGAA-3’
	Reverse	5’-GGTTGCCCAATGAATACTGC-3’

Supplementary Table 1. Primer sequences used in the study.

Gene name		Primer sequence
Human *PPARGC1A*	Forward	5'-CCAAAGGATGCGCTCTCGTTCA-3'
	Reverse	5'-CGGTGTCTGTAGTGGCTTGACT-3'
Human *TFAM*	Forward	5'-GTGGTTTTCATCTGTCTTGGCAAG-3'
	Reverse	5'-TTCCCTCCAACGCTGGGCAATT-3'
Human *NRF1*	Forward	5'-GGCAACAGTAGCCACATTGGCT-3'
	Reverse	5'-GTCGTCTGGATGGTCATCTCAC-3'
Human *NFE2L2*	Forward	5'-CACATCCAGTCAGAAACCAGTGG-3'
	Reverse	5'-GGAATGTCTGCGCCAAAAGCTG-3'
Mouse *Ppargc1a*	Forward	5'-AAGTGGTGTAGCGACCAATCG-3'
	Reverse	5'- AATGAGGGCAATCCGTCTTCA-3'
Mouse *Tmem26*	Forward	5'- ACCCTGTCATCCCACAGAG-3'
	Reverse	5'- TGTTTGGTGGAGTCCTAAGGTC-3'
Mouse *Dio2*	Forward	5'- GGTGGTCAACTTTGGTTCAGCC-3'
	Reverse	5'- AAGTCAGCCACCGAGGAGAACT-3'
Mouse *Cidea*	Forward	5'- GCAACCAAAGAAATCGGGAATA-3'
	Reverse	5'- GGTTACATGAACCAGCCTTTG-3'
Mouse *Kcnk3*	Forward	5'- GGCTCCTTCTACTTCGCCATCA-3'
	Reverse	5'- TGTTGATGCGTTCACCCAGGCT-3'
Mouse *Alpl/Tnap*	Forward	5'- CCAGAAAGACACCTTGACTGTGG-3'
	Reverse	5'- TCTTGTCCGTGTCGCTCACCAT-3'
Mouse *Atp1a2*	Forward	5'- ACAGGAACCCTAAGGTGGCAGA-3'
	Reverse	5'- CGCCTTTCATCACCAGCACATG-3'
Mouse *Ckb*	Forward	5'- GCTCATTGACGACCACTTCCTC-3'
	Reverse	5'- CCTCCTCGTTAATCCACACCAG-3'
Mouse *Atp2a2/Serca2*	Forward	5'- GTGAAGTGCCATCAGTATGACGG-3'
	Reverse	5'- GTGAGAGCAGTCTCGGTAGCTT-3'

Supplementary Table 1. Primer sequences used in the study (continued).

Compound	[1]Fold change	[2]Targets
Daunorubicin hydrochloride	33.07	ADC Cytotoxin; Antibacterial; Antibiotic; Apoptosis; Autophagy; DNA/RNA Synthesis; Topoisomerase
SP-8356	11.71	Autophagy
Ro 31-8220 Mesylate	6.19	PKC
MKT077	6.05	HSP
IT603	5.95	NF-κB
Emodin	3.48	Autophagy; Casein Kinase; SARS-CoV
NQ301	3.160616	Platelet aggregation; Thrombin
Ruboxistaurin hydrochloride	1.910787	PKC
SU5402	1.761219	FGFR; PDGFR; VEGFR
C188-9	1.746605	Apoptosis; STAT
MRX-2843	1.738897	FLT
Z-VAD(Ome)-FMK	1.687288	Caspase

Supplementary Table 2. High-throughput screening with the pre-clinical compound.
List of the 12 compounds showing over 1.5 fold of induction of TdTomato fluorescence intensity in *LETMD1* reporter beige adipocytes by screening the pre-clinical compound library.

[1.] Post/pre-dose signal values were first calculated for each well; The mean fold change of post/pre-dose signal values upon treatment relative to the in-plate DMSO control was then calculated for each compound and is presented in the list.

[2.] Reported main targets for these compounds.
